# Diastereoselectivity in the Staudinger reaction of pentafluorosulfanylaldimines and ketimines

**DOI:** 10.3762/bjoc.9.303

**Published:** 2013-11-27

**Authors:** Alexander Penger, Cortney N von Hahmann, Alexander S Filatov, John T Welch

**Affiliations:** 1 Department of Chemistry, University at Albany, SUNY, 1400 Washington Ave., Albany, NY 12222, USA

**Keywords:** aldimine, Cornforth transition state, diastereoselectivity, β-lactam, organo-fluorine, α-pentafluorosulfanyl aldehyde

## Abstract

β-Lactams were diastereoselectively formed by the reaction of SF_5_-containing aldimines, or an SF_5_-containing ketimine, with benzyloxyketene in a conrotatory ring closure process. Imine formation and cyclization were possible in spite of the acidification of protons on the carbon bound to SF_5_. The reactions of the aldimines demonstrated very good 1,*2-lk* diastereoselectivity, however lack of stereochemical control of the C*–*N ketimine geometry was reflected in the stereochemistry of the product β-lactam. Cyclization of imines with a stereogenic center bearing SF_5_ was reflected in the 1,2-*lk,lk* selectivity of the β-lactam.

## Introduction

The pentafluorosulfanyl (SF_5_) group is one of the few truly new functional groups to be introduced to synthetic organic chemistry in the last 100 years [1–6]. With pseudooctahedral geometry around sulfur, the SF_5_ group is a unique substituent for the medicinal or pharmaceutical chemist. While the electron withdrawing properties of the SF_5_ and CF_3_ groups are similar [7–8], the electronegativity of the SF_5_ group has been suggested to be as high as 3.65 in contrast to 3.36 for CF_3_ [9]. The inductive σ_I_ and σ_R_ values for SF_5_, 0.55 and 0.11 [10], contrast with σ_I_ and σ_R_ values for CF_3_ of 0.39 and 0.12 [11–12] illustrating the increased inductive effect of the SF_5_ group relative to the CF_3_ group. This effect can also be seen in the calculated dipole moments of 1,1,1-trifluoroethane and pentafluorosulfanylmethane of 2.589 and 3.556 Debye respectively [7–8]. When these electronic effects are combined with an occupied volume only slightly less than that of a *tert*-butyl group [3,13], the SF_5_ group can have unanticipated influences on the structure such as anchoring side chain and neighboring hydroxy group conformations [14–15].

Given the unique potential of the SF_5_ group, the rarity of its application in medicinal or agrochemical materials may be surprising. However, synthesis of aliphatic SF_5_-containing building blocks (SF_5_-substituted aromatic compounds are more accessible) [1,6], is challenging and often beset by confusing reactivity, largely because of the very properties that make SF_5_ an attractive substituent. Studies of aliphatic compounds have lagged as a consequence, a fact that is compounded by the lack of commercially available aliphatic SF_5_-containing building blocks. Although the number of known aliphatic pentafluorosulfanylated compounds is constantly increasing [2], this expansion has not generally been accompanied by applications of these materials. Among aliphatic SF_5_-substituted compounds α-pentafluorosulfanylated aliphatic carbonyl compounds [16], readily prepared from the corresponding enol acetates or enol ethers, are especially valuable as starting materials [17–22]. In these compounds the profound steric influence of the SF_5_ group [14–15] is accompanied by a dramatic increase in the acidity of the adjacent α-proton, a phenomenon underlying the abstraction of protons by methyllithium and a number of different ylides [16]. The reactions of α-pentafluorosulfanyl carbonyl compounds are governed by a combination of the substantial dipole moment and unique steric effects of the octahedral SF_5_ group. Aliphatic SF_5_-containing derivatives of biologically active compounds are not well-known. One exception is the inclusion of an ω-SF_5_-substituted amino acid incorporated at the crucial first and fourth positions of a heptapeptide [23]. When introduced at these positions, the SF_5_-substituted amino acid had a strong propensity to direct α-helix formation of even this short heptapeptide presumably by minimization of unfavourable hydrophobic interactions.

## Results and Discussion

The use of fluoroalkylimines to form β-lactams has proven especially useful in synthesis [24–26] especially in the Ojima β-lactam synthon method [27–29] used to prepare docetaxel analogs [26]. The general utility of the familiar Staudinger reaction of imines to transform readily accessible aldehydes to β-lactams has been well reviewed [30–33], yet in spite of this familiarity, the mechanism of this process remains a topic of interest [34–36].

Previously, it has been shown that fluorinated imines can undergo a manifold of reactions that are difficult to access with fluorinated aldehydes or ketones [37–40]. While there are many reports of the utility of trifluoroacetaldimines [41–44], there is only a single report of the preparation of *N-*ethyl 3,3,3-trifluoropropanaldimine [45], the trifluoromethyl analog of pentafluorosulfanylacetaldehyde (**1a**). The imine (3,3,3-trifluoropropanaldimine), in combination with the isomeric trifluoropropenamine, was not formed by the condensation of an amine with the aldehyde but rather by addition of an amine to 3,3,3-trifluoropropyne. The imine was described as “extremely unstable” [45] with no selective reactions reported. In light of this precedence the reaction of α-SF_5_-substituted aldehydes and ketones with amines was particularly intriguing.

### α-SF_5_-Substituted aldehydes and ketones

**Scheme 1:**
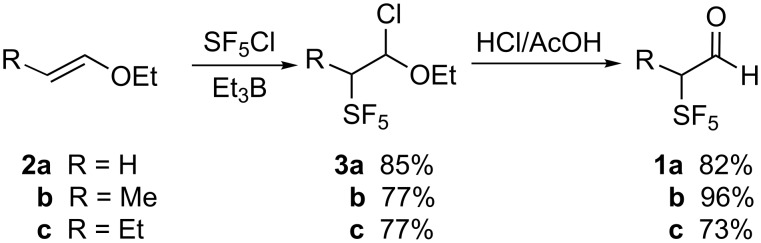
Synthesis of 2-pentafluorosulfanylaldehydes by addition of SF_5_Cl to enol ethers.

In this work the SF_5_-bearing aldehyde **1** was prepared by the addition of SF_5_Cl to the enol ether **2** instead of the previously described additions to enol acetates [16] (Scheme 1). In earlier studies, it was found that the yield of SF_5_Cl addition to enol acetates was highly dependent upon the purity of the enol acetate substrate, compounds surprisingly difficult to purify. Since vinyl acetate is the only enol acetate readily accessible for this reaction, the commercial availability of high purity propenyl and butenyl ethers **2b** and **2c** rendered these starting materials highly attractive for the formation of SF_5_-bearing aldehydes.

After addition of SF_5_Cl to **2**, intermediate **3** was typically formed as a 9:1 mixture of diastereomers. Hydrolysis of **3** was easily followed by ^19^F NMR, e.g., the resonance for **3b** appeared approximately 7 ppm upfield from that of the aldehyde **1b**. The *J*_H,F_ coupling constant of 5.0 Hz contrasts with the *J*_Feq,Fax_ value of 144 Hz.

The ready availability of the α-pentafluorosulfanyl carbonyl compounds facilitated an effort to dramatically expand the utility of these intriguing materials by synthesis and characterization of the corresponding pentafluorosulfanylated β-lactams.

### α-SF_5_-Substituted aldimines and ketimines

**Scheme 2:**
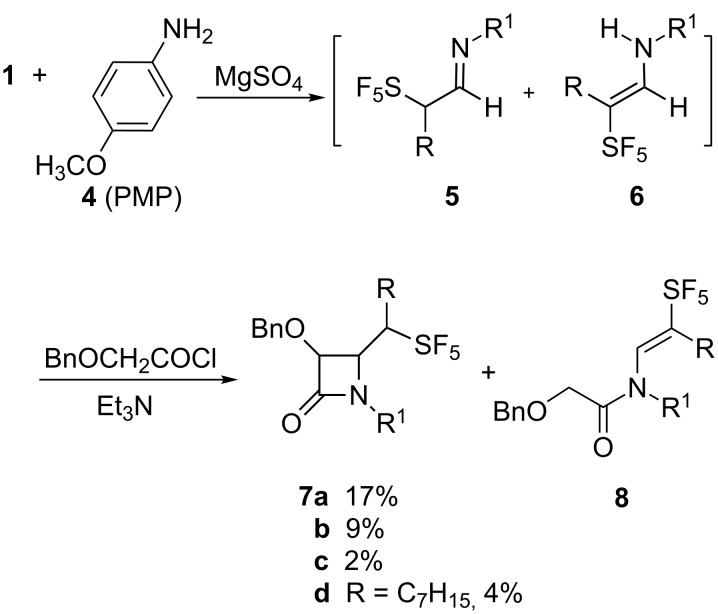
Reaction of pentafluorosulfanylaldimines with benzyloxyketene.

The aldehydes **1** were readily converted to the corresponding imine **5** in dichloromethane using anhydrous magnesium sulfate as a dehydrating agent (Scheme 2). Other desiccants, especially inherently basic materials, such as potassium carbonate lead to little imine formation. While the crude imine solution likely contained unreacted amine, attempted separation by silica gel chromatography led to extensive decomposition. The product imine consisted of a single stereoisomer as determined by ^19^F NMR, tentatively assigned as the *E-*isomer. Similar to the formation of **1**, it was easy to follow formation of the imine by ^19^F NMR with the resonance attributed to the equatorial fluorines of the imine **5a** (δ 68.8 ppm) appearing upfield of those assigned to the aldehyde **1a** (δ 72.4 ppm). In the case of imines **5b–d**, the solution also contained between 15–20% of the putative enamine **6b–d**, where, for example, the equatorial fluorine resonances of **6b** appeared at δ 65.5 ppm in contrast to those of **5b** that appeared at δ 59.5 ppm. The tendency for the enamine resonances to appear downfield of imine resonances was confirmed by the preparation of the morpholine enamine of **1a** for which the equatorial fluorine resonance is also shifted downfield [46]. As mentioned above, the preparation of *N-*ethyl 3,3,3-trifluoropropanaldimine, the Schiff base of 3,3,3-trifluoropropanal, was accompanied by enamine formation, likely as a consequence of the acidity of the proton α to the trifluoromethyl group [45,47]. Not surprisingly, **5** was relatively reactive. After careful filtration of the desiccant, the dichloromethane solution of the imine was used in further reactions without purification or separation of unreacted amine or the enamine side product.

### Ketene imine cycloaddition reactions of α-SF_5_-substituted aldimines and ketimines

Dropwise addition of the crude dichloromethane solution of **5** to benzyloxyacetyl chloride and triethylamine in dichloromethane was completed at 0 °C. The solution was then allowed to warm to room temperature with stirring overnight. Not surprisingly, the use of the crude solution of the imine led to the formation of a complex mixture where the desired product β-lactam **7** was a minor constituent. However, the yields of **7** are of product purified by silica gel chromatography and crystallization. The only other fluorinated products observed prior to purification were unreacted imine **5** and the tentatively designated *N-*acylenamine **8.** In the case of **7b–d** the de of the 1,2-*lk* to 1,2-*ul* products was 76%, 84% and 50% respectively. The relative proportion of the product mixture that was comprised of enamine **8b–d** was 10%, 4% and 21% respectively.

In an effort to improve the reaction, the addition of triethylamine to a solution of the acid chloride and imine **5** resulted only in decomposition. Excess amine **4** that was present was acylated by benzyloxyacetyl chloride to form the corresponding amide.

**Scheme 3:**
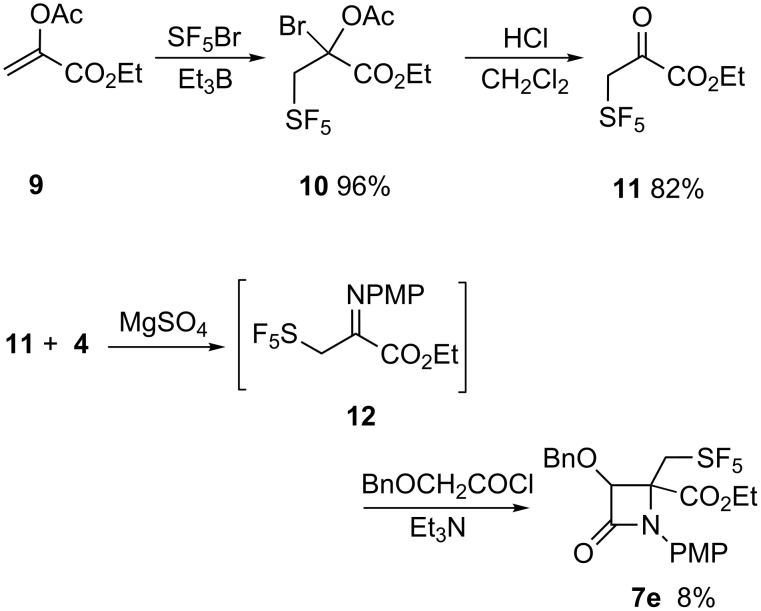
Preparation of ethyl pentafluorosulfanylpyruvate and formation of the corresponding β-lactam.

The utility of the ketene–imine cyclization was not limited to aldimines. The addition of SF_5_Br to the enol acetate of ethyl pyruvate **9** formed ethyl pentafluorosulfanylpyruvate **11** (Scheme 3). The ketimine **12**, prepared via amine condensation with **11,** was reacted as described for the aldimines **5** to form the desired β-lactam **7e**.

Formation of **7e** was accompanied by significantly greater decomposition of **12** and hence **7e** was formed in lower overall yields. In contrast to the stereoselective formation of **7b–d**, the diastereoselectivity of the Staudinger reactions as determined by ^19^F NMR was dramatically reduced for **7e** to a de of 14%, a value consistent with the *Z*/*E* ratio for **12** of 0.7. While the isolated yields of purified **7b–e** are especially modest, the reaction conditions have not been optimized. But the significance of these findings lies not only in the difference in reactivity in comparison with trifluoropropanaldimine but also in the relative diastereoselectivity of the ketene–imine cycloaddition reaction in comparison with the other reactions of SF_5_-bearing aldehydes [16].

### Structural characterization of SF_5_-containing β-lactams

**Figure 1:**
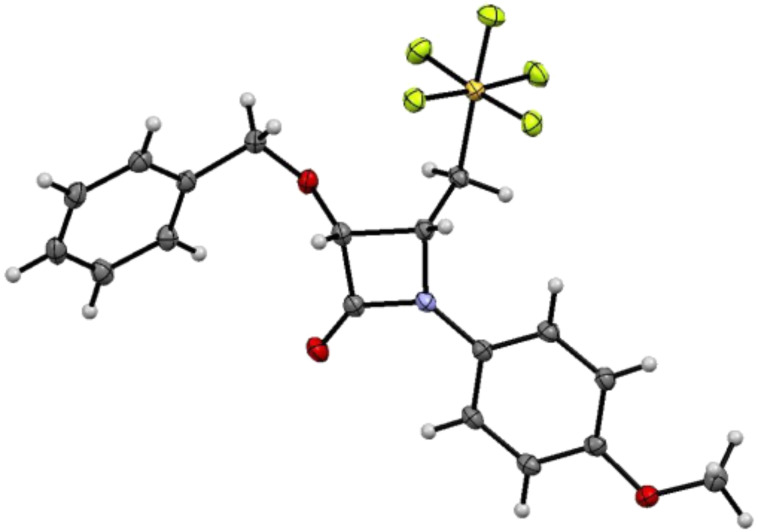
The 1,2-*lk* stereochemistry of **7a** as determined by single crystal X-ray diffraction. Thermal ellipsoids are set at 50% probability.

**Scheme 4:**
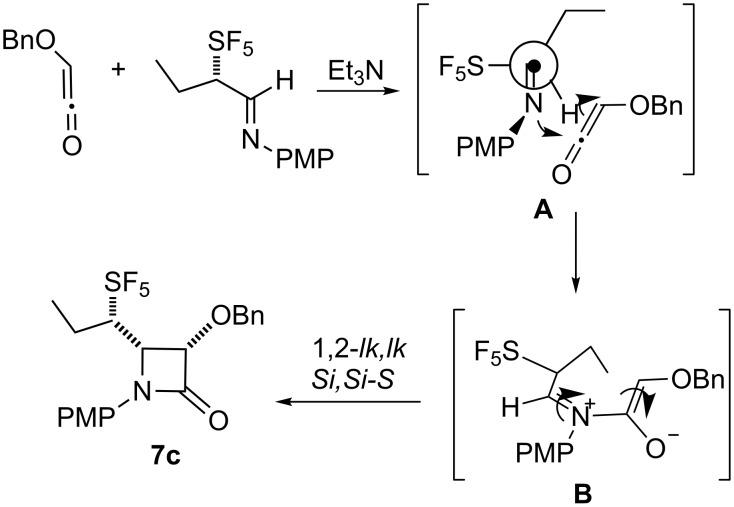
Influence of the SF_5_ group on the initial attack of the ketene on the imine nitrogen (**A**) and on the sense of conrotatory ring closure (**B**).

Isolated as a single diastereomer, the relative stereochemistry of **7a**, the product of the Staudinger reaction of **5a**, is shown in Figure 1. The *cis* relative stereochemistry of β-lactam is consistent with 1,2-*lk* conrotatory ring closure of the *E*-imine **5a** as would be predicted for a reaction with the Bose–Evans ketene formed from benzyloxyacetyl chloride [30]. The low yield of β-lactam product is better understood when the reactivity of the intermediate pentafluorosulfanylated imine and the subsequently formed iminium ion are considered. The SF_5_ group increases the acidity of the α-proton of the imine **5** and of the iminium ion intermediate **B** formed on the initial nucleophilic attack of the imine on the ketene as illustrated in Scheme 4. The ring closure step requires bond formation between the iminium ion carbon and the enolate carbon **B** to be particularly facile for the stereoselectivity of the process to be preserved. The ring closure process must compete successfully with loss of the acidic proton from **B** to form **8** [16]. Another indication of the rapidity of ring closure is the failure to detect the 1,2-*ul* product. The absence of 1,2-*ul* product is consistent with retardation the rate of *E*/*Z* imine isomerization by the electron withdrawing pentafluorosulfanyl group [35]. In the reaction of **12**, the *E*/*Z* ratio of the imine was reflected very well in the 1,2-diastereomeric excess of the product β-lactam **7e** .

**Figure 2:**
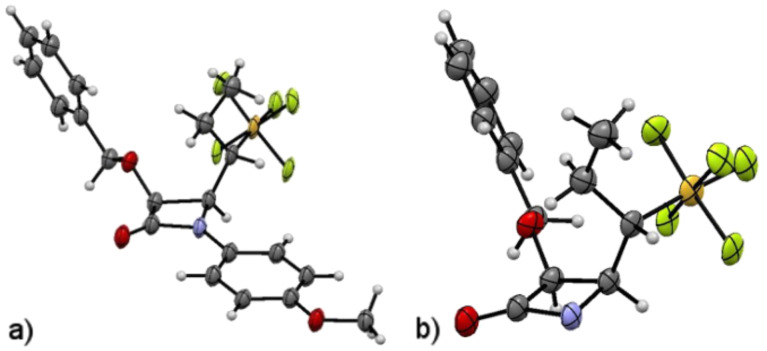
The stereochemistry of **7c,** 1,2-*lk,lk* (*Si, Si-S*), as determined by single crystal X-ray diffraction studies. Thermal ellipsoids are set at 50% probability. (a) Complete structure of **7c**. (b) PMP protecting group hidden for clarity.

The ketene–imine condensation of **7c** is influenced by the presence of the pentafluorosulfanyl group at a stereogenic center. The 1,2-*lk,lk* (*Si,Si-S*) (or (*Re,Re-R*)) stereochemistry of **7c** (Figure 2) suggests the profound dipole associated with the introduction of the SF_5_ group may influence the diastereoselectivity of ring closure. The initial approach of the ketene (**A** in Scheme 4) appears to be influenced by avoidance of unfavorable interaction of the ketene with the sterically demanding SF_5_ group. Previously it was found in single crystal X-ray diffraction studies that the pentafluorosulfanyl group [48] is predictably orthogonal to a carbonyl group as shown in **A**. Cornforth control of the ring closure step of the zwitterion (**B** in Scheme 4) where the SF_5_ would be antiperiplanar to the iminium ion would lead to the observed diastereoselectivity (Scheme 1). This finding is consistent with dipolar effects being most important in reactions with highly charged transition states such as **B**.

In both structures, consistent with the opposing dipole geometry of the Cornforth transition state, the N–C–C–S torsional angles remain near 170° (169° and 167° for **7a** and **7c** respectively).

The 1,2-*lk,ul* ring closure product may be formed in the reaction of **5c** with benzyloxyketene and simply remain undetected, but it is clear that the control of diastereofacial selectivity in the formation of the principal β-lactam is strongly under the control of the SF_5_ group.

## Conclusion

Low molecular weight pentafluorosulfanylated aldehydes **1** were prepared by addition of SF_5_Cl to enol ethers and the subsequent acidic hydrolysis of **3**. Formation of Schiff base **5** is problematic but, in contrast to the reactions of the analogous trifluoromethyl compounds, does successfully proceed. Even with a manifold of possible side reactions, β-lactam formation by the ketene–imine cycloaddition reaction of **5** occurs, albeit in very modest yields. The 1,2-*lk* stereochemistry of the β-lactam **7** was consistent with rapid cyclization and a failure of the imines **5** to isomerize. The presence of a pentafluorosulfanylated stereogenic carbon as in **5c**, apparently also influences the 2,3-*lk* stereochemistry. Optimization of β-lactam synthesis will require a better understanding of the nature of the competing, undesirable reactions and enable utilization of this unique construct in further synthetic transformations. The product β-lactams are a useful entrée to the diastereoselective synthesis of pentafluorosulfanyl β-amino acids and suggest a path to the preparation of more extensively functionalized SF_5_-containing β-lactams.

## Supporting Information

File 1 Detailed experimental procedures and spectroscopic data for **1a–e**, **10**, **5a–d**, **7a–e** and **11**.

File 2 X-ray crystallographic data for **7a** and **7c,** CCDC 937908 and 937909.
