# Supplementary material for: Diastereoselectivity in the Staudinger reaction of pentafluorosulfanylaldimines and ketimines
Source: Beilstein J Org Chem. 2013 Nov 27;9:2675–80. doi: 10.3762/bjoc.9.303 (PMC3869290; doi:10.3762/bjoc.9.303)
Supplement: File 1 — Detailed experimental procedures and spectroscopic data for 1a–e, 10, 5a–d, 7a–e and 11. [file Beilstein_J_Org_Chem-09-2675-s001.pdf]

Supporting Information

for

Diastereoselectivity in the Staudinger reaction of pentafluorosulfanylaldimines and ketimines

Alexander Penger, Cortney N. von Hahmann, Alexander S. Filatov and John T. Welch*

Address: Department of Chemistry, University at Albany, SUNY, 1400 Washington Ave., Albany, NY 12222, USA

Email: John T. Welch - jwelch@albany.edu

*Corresponding author

Detailed experimental procedures and spectroscopic data for 1a–e, 10, 5a–d, 7a–e and 11

Contents

General methods S2

Experimental details and spectroscopic data

(**1a–d**) S2

(**11**) S4

(**5a–d**) S5

(**7a–e**) S7

(**12**) S9

Crystallography S11

Presentation of NMR spectra

(**1a**) 2-Pentafluorosulfanylacetaldehyde S13

(**1b**) 2-Pentafluorosulfanylpropanal S14

(**1c**) 2- Pentafluorosulfanylbutanal S15

(**1d**) 2- Pentafluorosulfanylnonanal S16

(**11**) Ethyl 3-pentafluorosulfanyl-2-oxopropanoate S17

(**5a**) *N*-(2-Pentafluorosulfanylethylidene)-4-methoxybenzenamine S18

(**5b**) *N*-(2-Pentafluorosulfanylpropylidene)-4-methoxybenzenamine S19

(**5c**) *N*-(2-Pentafluorosulfanylbutylidene)-4-methoxybenzenamine S20

(**5d**) *N*-(2-Pentafluorosulfanylnonylidene)-4-methoxybenzenamine S21

(**7a**) (3*SR*,4*RS*)-3-(benzyloxy)-4-(pentafluorosulfanylmethyl)-1-(4-methoxyphenyl)azetidin-2-one S23

(**7b**) (3*SR*,4*RS*)-3-(Benzyloxy)-4-((1'*SR*)-1-pentafluorosulfanyl-ethyl)-1-(4-methoxyphenyl)azetidin-2-one S25

(**7c**) (3*SR*,4*RS*)-3-(Benzyloxy)-4-((1'*SR*)-1-pentafluorosulfanyl-propyl)-1-(4-methoxyphenyl)azetidin-2-one S27

(**7d**) (3*SR*,4*RS*)-3-(Benzyloxy)-4-((1'*SR*)-1-pentafluorosulfanyl-octyl)-1-(4-methoxyphenyl)azetidin-2-one S29

(**7e**) Ethyl 3-(benzyloxy)-2-(pentafluorosulfanylmethyl)-1-(4-methoxyphenyl)-4-oxoazetidine-2-carboxylate S31

General methods:

All reactions were carried out under an inert atmosphere. Reagents were purchased from commercial sources and used without further purification. All solvents were purified by standard methods and freshly distilled under argon. NMR spectra were recorded in a 400 MHz spectrometer: ^{1}H (400 MHz), ^{13}C (100 MHz), and ^{19}F (376 MHz). The chemical shifts of ^{1}H and ^{13}C NMR are reported relative to the residual signal of $CDCl_3$ (for ^{1}H: δ = 7.24; ^{13}C: δ = 77.00) or C_6D_6 (for ^{1}H: δ = 7.15; ^{13}C: δ = 128.00). All ^{13}C NMR spectra were acquired in the proton-decoupled mode. ^{19}F NMR spectra are reported relative to the resonance assigned to $CFCl_3$ (δ = 0). Thin layer chromatography was performed with silica gel F_{254} as the adsorbent on 0.2 mm thick, plastic-backed plates. The chromatograms were visualized under UV (254 nm) or by staining with a $KMnO_4$ aqueous solution followed by heating. Column chromatography was performed using

silica gel 60 (70–230 mesh). HRMS analyses were performed by the Proteomics Core Facility of the Center for Functional Genomics of the University at Albany.

2-Pentafluorosulfanylaldehydes

General procedure for preparation of 3a–c. To a solution of pentafluorosulfanyl chloride (2 mmol, 0.9 M in pentane) at −40 °C was added the enol ether **2** (1.54 mmol, 1 equiv) dissolved in 1 mL of pentane. After 5 min, this mixture was aerated by the bubbling of 2–3 mL of air by syringe, immediately after aeration 0.4 mL of triethylboron (1 M solution in hexanes, 0.1 equiv) in hexane was added via syringe pump over 30 min. The mixture was allowed to warm to rt with stirring over 1.5 h and then quenched with saturated $NaHCO_3$ solution. The mixture was then extracted with dichloromethane, the organic fractions washed with brine, dried ($MgSO_4$) and filtered and concentrated. The crude product was subjected to hydrolysis without further purification.

Preparation of 1a–c from 3: A mixture of the 1-chloro-2-pentafluorosulfanylalkyl ether (1–10 mmol), aqueous HCl (3.5 M, 9 mL) and glacial acetic acid (6 mL) was heated to 50 °C overnight. After cooling to room temperature, dichloromethane was added and the mixture poured into a beaker containing 40 mL of 20% Na_2CO_3. The aqueous layer was extracted with dichloromethane (2×) and the combined organic fractions washed with water (2×), brine, dried with Na_2SO_4, and filtered. The product was concentrated by cautious distillation of the dichloromethane by spinning band distillation, 10:1 reflux ratio, at ambient pressure.

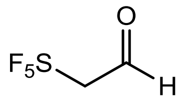

(**1a**) 2-Pentafluorosulfanylacetaldehyde (yield 82%)

^{1}H-NMR ($CDCl_3$) δ/ppm: 9.79-9.77 (m, 1H, CHO), 4.37 (pd, 2H, ^3J(H-F) = 8.0 Hz, ^3J(H-H) = 2.5 Hz, CH_2).

^{19}F-NMR ($CDCl_3$) δ/ppm: 80.4 (9 signals, 1F), 72.4 (dm, 4F, ^2J(F-F) = 147.9 Hz).

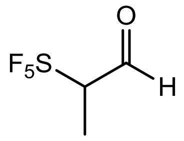

(**1b**) 2-Pentafluorosulfanylpropanal (yield 96%)

^{1}H-NMR ($CDCl_3$) δ ppm: 9.80-9.77 (m, 1H, CHO), 4.52-4.43 (m, 1H, CH), 1.64 (dp, ^3J(H-H) = 7.2 Hz, ^4J(H-F) = 2.8 Hz, CH_3).

^{19}F-NMR ($CDCl_3$) δ/ppm: 82.1 (9 signals, 1F), 62.7 (dd, 4F, ^2J(F-F) = 144.6 Hz, ^3J(F-H) = 5.4 Hz).

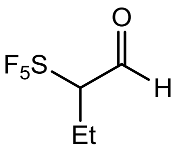

(**1c**) 2- Pentafluorosulfanylbutanal (yield 73%)

^{1}H-NMR ($CDCl_3$) δ/ppm: 9.67-9.63 (m, 1H, CHO), 4.26-4.17 (m, 1H, CH), (m, CH_2), 1.01 (t, 3H, ^{3}J=7.5 Hz, CH_3).

^{19}F-NMR ($CDCl_3$) δ/ppm: 82.6 (9 signals, 1F), 64.3 (dd, 4F, ^{2}J(F-F) = 144.6 Hz, ^{3}J(F-H) = 6.0 Hz).

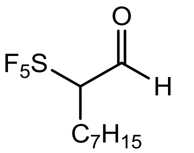

(**1d**) 2- Pentafluorosulfanylnonanal[1]

^{1}H-NMR ($CDCl_3$) δ/ppm: 9.66-9.61 (m, 1H, CHO), 4.33-4.21 (m, 1H, H_α), 2.21-2.12 (m, 2H, H_β), 1.39-1.21 (m, 10H), 0.92-0.87 (m, 3H).

^{19}F-NMR ($CDCl_3$) δ/ppm: 82.7 (9 signals, 1F), 64.2 (dd, 4F, ^{2}J(F-F) = 144.6 Hz, ^{3}J(F-H) = 5.8 Hz).

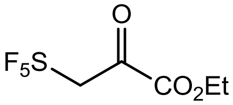

(**11**) Ethyl 3-pentafluorosulfanyl-2-oxopropanoate (yield 79%)

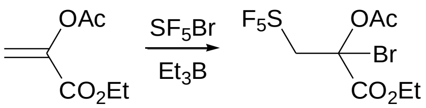

Into a mixture of pentafluorosulfanyl bromide (0.4 M in pentane, 5 mL) and triethylboron (1 M in hexane, 0.2 mL) was added the enol acetate of ethyl pyruvate (237 mg, 1.5 mmol in 1 mL pentane) at 0 °C. The reaction mixture was stirred for 15 min then an additional portion of triethylboron (0.1 mL) was added. The mixture was stirred for another 15 min and then was quenched with sat. $NaHCO_3$. Following extraction with diethyl ether and driying with anhydrous $MgSO_4$, concentration in vacuo gave the crude product (525 mg, 96%) which was used in subsequent transformation without purification.

^{1}H NMR ($CDCl_3$) δ 5.13-5.00 (m, 1H), 4.79-4.67 (m, 1H), 4.46 – 4.31 (m, 2H), 2.24 (s, 3H), 1.37 (t, *J* = 7.2 Hz, 3H).
^{19}F NMR ($CDCl_3$) δ 80.30 (p, *J* = 149.0, 1F), 68.66 – 68.12 (dt, *J* = 149.0, 7.3 Hz, 4F).

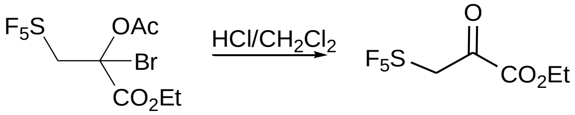

To a solution of the bromoacetate (960 mg, 2.6 mmol) in CH_2Cl_2 (10 mL) was added 1 drop of HCl (3.5 M in water) and 1 drop of water. The reaction mixture was heated under reflux overnight. After cooling to rt and drying with K_2CO_3 for 20 min, concentration in vacuo gave the crude product (518 mg, 82%).

^{1}H NMR (400 MHz, $CDCl_3$) δ 4.82 (p, *J* = 7.6 Hz, 1H), 4.42 (q, *J* = 7.2 Hz, 2H), 1.41(t, *J* = 7.2 Hz, 3H).
^{19}F NMR (376 MHz, $CDCl_3$) δ 78.72 (p, *J* = 151.8, 1F), 68.66 – 68.12 (dm, *J* = 151.8 Hz, 4F).

General procedure for imine synthesis

To a flame dried round-bottom flask was added 5 equivalents of anhydrous magnesium sulfate followed by dry, distilled dichloromethane, to this suspension protected from atmospheric moisture was added a dichoromethane solution of the aldehyde (0.43 M). A dichloromethane solution (0.43 M) of the desired amine **6** was then added dropwise with stirring. Removal of the solids by filtration in the absence of atomospheric moisture, yielded a dichloromethane solution containing imine **7** that was utilized directly in the Staudinger reaction.

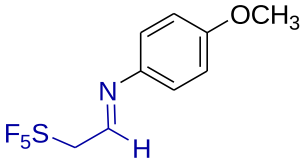

(**5a**) *N*-(2-Pentafluorosulfanylethylidene)-4-methoxybenzenamine
^{1}H-NMR ($CDCl_3$) δ/ppm: 8.00-7.95 (m, 1H), 7.15 (d, 2H, ^3J = 8.9 Hz), 6.91 (d, 2H, ^3J = 8.9 Hz), 4.67-4.57 (m, 2H, CH_2), 3.82 (s, 3H, CH_3).

^{19}F-NMR ($CDCl_3$) δ/ppm: 81.3 (9 signals, 1F), 68.8 (dt, 4F, ^2J(F-F) = 146.1 Hz, ^3J(F-H) = 7.3 Hz).

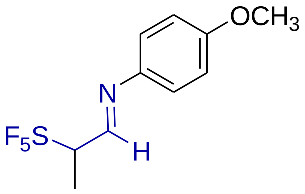

(**5b**) *N*-(2-Pentafluorosulfanylpropylidene)-4-methoxybenzenamine

^{1}H-NMR ($CDCl_3$) δ/ppm: 7.97-7.93 (m, 1H), 7.12 (d, 2H, 3J = 8.8 Hz), 6.91 (d, 2H, 3J = 8.8 Hz), 4.83-4.74 (m, 1H, CH), 3.82 (s, 3H, OCH_3), 1.77 (dp, 3H, ^{3}J(H-H) = 7.1 Hz, CH_3).

^{19}F-NMR ($CDCl_3$) δ/ppm: 83.0 (9 signals, 1F), 64.5 (enamine) (d, 4F, ^{2}J(F-F) = 148.6 Hz), 58.7 (dd, 4F, ^{2}J(F-F) = 143.0 Hz, ^{3}J(F-H) = 4.4 Hz).

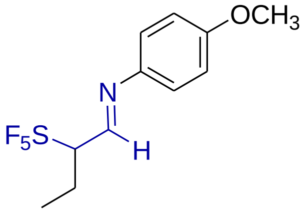

(**5c**) *N*-(2-Pentafluorosulfanylbutylidene)-4-methoxybenzenamine

^{1}H-NMR ($CDCl_3$) δ/ppm: 7.84-7.78 (m, 1H), 7.14 (d, 2H, ^{3}J = 8.4 Hz), 6.92 (d, 2H, ^{3}J = 8.4 Hz), 4.61-4.50 (m, 1H, CH), 3.82 (s, 3H, OCH_3), 2.42-2.32 (m, 1H, CH_2), 2.26-2.16 (m, 1H, CH_2), 1.02 (t, 3H, ^{3}J = 7.3 Hz, CH_3).

^{19}F-NMR ($CDCl_3$) δ/ppm: 83.5 (9 signals, 1F), 65.8 (enamine) (d, 4F, ^{2}J(F-F) = 148.7 Hz), 59.7 (dd, 4F, ^{2}J(F-F) = 143.3 Hz, ^{4}J(F-H) = 4.7 Hz).

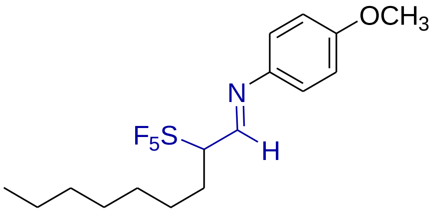

(**5d**) *N*-(2-Pentafluorosulfanylnonylidene)-4-methoxybenzenamine

^{1}H-NMR ($CDCl_3$) δ/ppm: 7.79 (m, 1H, ^{3}J = 6.4 Hz), 7.13 (d, 2H, ^{3}J = 8.8 Hz), 6.92 (d, 2H, ^{3}J = 8.6 Hz), 4.67-4.56 (m, 1H, H_α), 3.83 (s, 3H), 1.41-1.21 (m, 10H), 0.93-0.84 (m, 3H).

^{19}F-NMR ($CDCl_3$) δ/ppm: 83.5 (9 signals, 1F), 65.7 (enamine) (d, 4F, ^{2}J(F-F) = 148.8 Hz), 59.6 (dd, 4F, ^{2}J(F-F) = 143.1 Hz, ^{3}J(F-H) = 5.1 Hz).

General procedure for β-lactam synthesis.

In a typical procedure, to a dichloromethane solution of triethylamine (0.537 g, 5.37 mmol, 3 mL) was added benzyloxyacetyl chloride (0.978 g, 5.3 mmol) dissolved in another 3 mL of dichloromethane. The imine **5** (1.28 mmol) in 4.5 mL of dichloromethane was added dropwise to the previously prepared solution of triethylamine and benzyloxyacetyl chloride at 0 °C. The reaction mixture was allowed to warm to room temperature and to stir overnight. The reaction was quenched with ca 6 mL of saturated $NaHCO_3$, following separation of the phases, the aqueous phase was extracted three times with dichloromethane. The combined organic extracts were dried over $MgSO_4$, filtered and concentrated in vacuo. The desired β-lactam **7** was isolated by column chromatography using 4:1, ethyl acetate/hexanes.

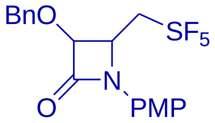

(**7a**) (3*SR*,4*RS*)-3-(Benzyloxy)-4-(pentafluorosulfanylmethyl)-1-(4-methoxyphenyl)azetidin-2-one (yield 17%)

^{1}H-NMR ($CDCl_3$) δ/ppm: 7.44-7.27 (m, 7H, H_{Bn}, H_{PMP}), 6.95-6.90 (m, 2H, H_{PMP}), 4.90 (AB, 2H, ^2J = 11.7 Hz, H_{Bn}), 4.90 (d, 1H, ^3J = 4.8 Hz, H_γ), 4.82 (ddd, 1H, ^3J = 8.7 Hz, ^3J = 4.8 Hz, ^3J = 1.2 Hz, H_β), 4.43-4.29 (m, 1H, H_α), 4.05-3.91 (m, 1H, H_α), 3.80 (s, 3H, OCH_3).

^{13}C{^{1}H}-NMR ($CDCl_3$) δ/ppm: 163.4 (<u>C</u>=O), 157.0, 136.5, 128.8, 128.6, 128.2, 127.9, 118.6, 114.9, 80.8 (C_γ), 73.6, 65.3 (^2J(C-F) = 13.0 Hz, C_α), 55.5, 53.9 (^3J(C-F) = 5.9 Hz, C_β).

^{19}F-NMR ($CDCl_3$) δ/ppm: 83.3 (9 signals, 1F 146.7 Hz), 66.9 (dt, 4F, ^2J(F-F) = 146.7 Hz, ^3J(F-H) = 8.0 Hz).

HRMS (ESI, positive): Exact mass calcd. $C_{18}H_{18}F_5NO_3S$ $[M]^+$ requires *m*/*z*: 423.0922, found *m*/*z*: 423.0912.

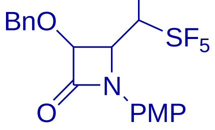

(**7b**) (3*SR*,4*RS*)-3-(Benzyloxy)-4-((1’*SR*)-1-pentafluorosulfanylethyl)-1-(4-methoxyphenyl)azetidin-2-one (yield 9%)

^{1}H-NMR ($CDCl_3$) δ/ppm: 7.43-7.27 (m, 7H, H_{Bn}, H_{PMP}), 6.94-6.89 (m, 2H, H_{PMP}), 4.94 (d, 1H, ^3J = 5.0 Hz, H_γ), 4.90 (dd, 1H, ^3J = 5.2 Hz, ^3J = 3.3 Hz, $H_{\beta\text{-ring}}$), (4.87 (AB, 2H, ^2J

= 11.7 Hz, H_{Bn}), 4.61-4.49 (m, 1H, H_{α}), 3.80 (s, 3H, OCH_3), 1.68 (d, 3H, ^3J = 6.9 Hz, H_{β}).

$^{13}C\{^1H\}$-NMR ($CDCl_3$) δ/ppm: 164.6 (C=O), 157.0, 136.7, 128.8, 128.5, 128.1, 127.8, 118.8, 114.9, 82.6, 79.4 (m), 74.3, 56.9 (m), 55.5, 12.8.

^{19}F-NMR ($CDCl_3$) δ/ppm: 84.6 (9 signals, 1F, 143.7 Hz), 57.9 (dd, 4F, ^2J(F-F) = 143.7 Hz, ^3J(F-H) = 6.3 Hz).

HRMS (ESI, positive): Exact mass calcd. $C_{19}H_{20}F_5NO_3S$ $[M]^+$ requires *m/z*: 437.1079, found *m/z*: 437.1071.

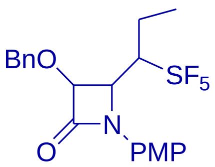

(**7c**) (3*SR*,4*RS*)-3-(Benzyloxy)-4-((1'*SR*)-1-pentafluorosulfanylpropyl)-1-(4-methoxyphenyl)azetidin-2-one (yield 2%)

^1H-NMR ($CDCl_3$) δ/ppm: 7.43-7.27 (m, 7H, H_{Bn}, H_{PMP}), 6.95-6.89 (m, 2H, H_{PMP}), 4.95 (dd, 1H, ^3J = 5.0 Hz, ^3J = 2.9 Hz, $H_{\beta\text{-ring}}$), 4.92 (d, 1H, ^3J = 5.0 Hz, $H_{\gamma\text{-ring}}$), 4.86 (AB, 2H, ^2J = 11.8 Hz, H_{Bn}), 4.41-4.30 (m, 1H, H_{α}), 3.80 (s, 3H, OCH_3), 2.54-2.40 (m, 1H, H_{β}), 2.17-2.04 (m, 1H, H_{β}), 0.96 (t, 3H, ^3J = 7.4 Hz, H_{γ}).

$^{13}C\{^1H\}$-NMR ($CDCl_3$) δ/ppm: 164.7 (C=O), 156.9, 136.8, 129.1, 128.5, 128.1, 127.8, 118.6, 114.9, 86.9 (m, C_{α}), 82.6 ($C_{\gamma\text{-ring}}$), 74.2, 57.6 (m, $C_{\beta\text{-ring}}$), 55.5, 21.2 (m, C_{β}), 13.7 (m, C_{γ}).

^{19}F-NMR ($CDCl_3$) δ/ppm: 85.9 (9 signals, 1F, ^2J(F-F) = 143.7 Hz) 60.9 (d, 4F, ^2J(F-F) = 143.6 Hz).

HRMS (ESI, positive): Exact mass calcd. $C_{20}H_{22}F_5NO_3S$ $[M]^+$ requires *m/z*: 451.1235, found *m/z*: 451.1219.

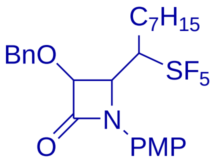

(**7d**) (3*SR*,4*RS*)-3-(Benzyloxy)-4-((1'*SR*)-1-pentafluorosulfanyloctyl)-1-(4-methoxyphenyl)azetidin-2-one (yield 4%)

^1H-NMR ($CDCl_3$) δ/ppm: 7.43-7.28 (m, 7H, H_{Bn}, H_{PMP}), 6.94-6.90 (m, 2H, H_{PMP}), 4.95 (dd, 1H, ^3J = 5.1 Hz, ^3J = 3.0 Hz, $H_{\beta\text{-ring}}$), 4.91 (d, 1H, ^3J = 5.1 Hz, $H_{\gamma\text{-ring}}$), 4.87 (AB, 2H, ^2J = 11.8 Hz, H_{Bn}), 4.49-4.39 (m, 1H, H_{α}), 3.80 (s, 3H, OCH_3), 2.48-2.36 (m, 1H, H_{β}), 2.09-1.96 (m, 1H, H_{β}), 1.12-1.02 (m, 6H), 0.94-0.84 (m, 6H), 0.81 (t, ^3J = 7.1 Hz).

$^{13}C\{^1H\}$-NMR ($CDCl_3$) δ/ppm: 164.7 (C=O), 156.9, 137.2, 128.6, 128.5, 128.1, 127.6, 118.6, 114.9, 82.5 (C_γ), 73.4, 67.2, 31.6, 29.7, 28.94, 28.64, 28.19, 27.5, 22.6, 14.03

^{19}F-NMR ($CDCl_3$) δ/ppm: 86.0 (9 signals, 1F, 2J(F-F) = 143.7 Hz), 60.7 (d, 4F, ^2J(F-F) = 143.8 Hz).

HRMS (ESI, positive): Exact mass calcd. $C_{25}H_{32}F_5NO_3S$ $[M]^+$ requires *m*/*z*: 521.2018, found *m*/*z*: 521.2013.

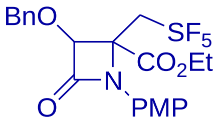

(**7e**) Ethyl 3-(benzyloxy)-2-(pentafluorosulfanylmethyl)-1-(4-methoxyphenyl)-4-oxoazetidine-2-carboxylate (yield 8%) Diastereomers designated A & B

^1H-NMR ($CDCl_3$) δ/ppm: 7.45-7.32 (m, 7H, H_{Bn}, H_{PMP}, A + B), 6.89-6.82 (m, 2H, H_{PMP}, A + B), 4.93 (s, 1H, $H_{\gamma,}$ A + B), 4.83 (AB, 2H, ^2J = 11.8 Hz, H_{Bn}, A + B), 4.52-4.05 (m, 4H, H_α, CH_2-ester, A + B), 3.80 (s, 3H, OCH_3, A), 3.79 (s, 3H, OCH_3, B), 1.30 (t, 3H, ^3J = 7.2 Hz, CH_3-ester, A), 1.25 (m, 3H, CH_3-ester, B).

$^{13}C\{^1H\}$-NMR ($CDCl_3$) δ/ppm: 168.6(C=O_{Ester} B), 168.5 (C=O_{Ester}, A), 164.5 (C=O_{Ring}, A), 163.9 (C=O, C_{Ring}, B), 158.5 (A), 157.3 (B), 136.2 (B), 135.6 (A), 129.2, 128.8, 128.73, 127.68, 128.5, 128.3, 128.1, 127.1, 125.9, 121.4, 114.2 (B), 114.0 (A), 88.0 (A), 86.0 (B), 74.7 (A), 74.5 (B), 71.3 (m), 69.4 (m), 69.2, 63.0 (A), 62.6 (B), 55.42 (B), 55.38 (B), 42.3, 14.0 (B), 13.9 (A).

^{19}F-NMR ($CDCl_3$) δ/ppm: 81.9 (9 signals, 1F, ^2J(F-F) = 149.9-145.2 Hz A + B), 71.2 (dt, 4F, ^2J(F-F) = 147.0, ^3J(F-H) = 8.9 Hz).

HRMS (ESI, positive): Exact mass calcd. $C_{21}H_{22}F_5NO_5S$ $[M]^+$ requires *m*/*z*: 495.1133, found *m*/*z*: 495.1163.

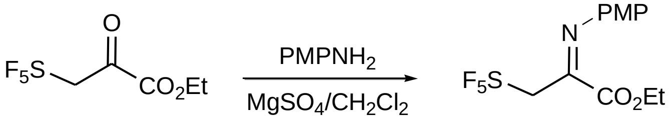

(**12**) (*E*)-ethyl 2-(4-methoxyphenylimino)-3-pentafluorosulfanylpropanoate

Procedure:

Into a solution of **11** (726 mg, 3 mmol) in DCM (15 mL) was added *p*-anisidine (1.10 g, 9 mmol) and $MgSO_4$ (1.8 g, 15 mmol). The reaction mixture was allowed to stir overnight. Filteration and concentration gave the crude product as a mixture of *E*/*Z*-isomers and unreacted of $PMPNH_2$.

Isomer 1: ^{1}H NMR (400 MHz, $CDCl_3$) δ 6.91 – 6.87 (m, 2H), 6.84 – 6.81 (m, 2H), 4.83 (p, *J* = 7.3 Hz, 2H), 4.13(q, *J* = 7.1 Hz, 1H), 3.81 (s, 3H), 1.06 (t, *J* = 7.1 Hz, 3H).

^{19}F NMR (376 MHz, $CDCl_3$) δ 80. 03 (p, *J* = 152.0 Hz, 1F), 70.49 (dm , *J* = 152.0 Hz, 4F).

Isomer 2: ^{1}H NMR (400 MHz, $CDCl_3$) δ 6.96 – 6.92 (m, 2H), 6.79 – 6.76 (m, 2H), 4.76 (p, *J* = 7.4 Hz, 2H), 4.47(q, *J* = 7.0 Hz, 1H), 3.83 (s, 3H), 1.43 (t, *J* = 6.9 Hz, 3H).

^{19}F NMR (376 MHz, $CDCl_3$) δ 80. 00 (p, *J* = 149.4 Hz, 1F), 69.50 (dm , *J* = 149.4 Hz, 4F).

Crystallography

The X-ray intensity data were collected for **7a** and **7c** on a Bruker APEX CCD X-ray diffractometer equipped with a graphite monochromated Mo Kα radiation source (λ = 0.71073 Å). Data reduction and integration were performed with the software package *SAINT*,[2] and absorption corrections were applied using the program *SADABS*.[3] All structures were solved and refined using the *SHELXTL* program package.[4] All non-hydrogen atoms were refined anisotropically. Hydrogen atoms were included at idealized positions as a riding model. Crystallographic data and X-ray experimental conditions for **7a** and **7c** are listed in Table S1.

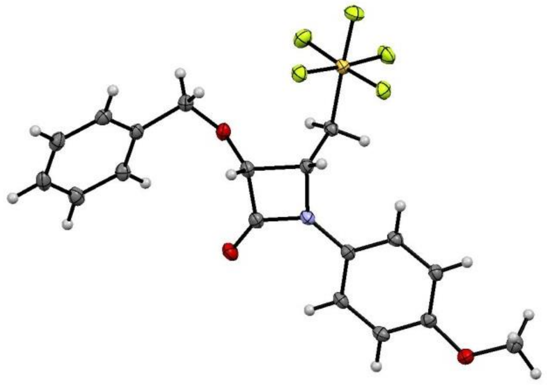

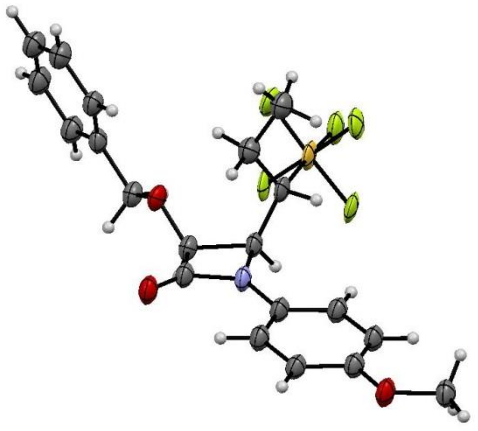

7a **7c**

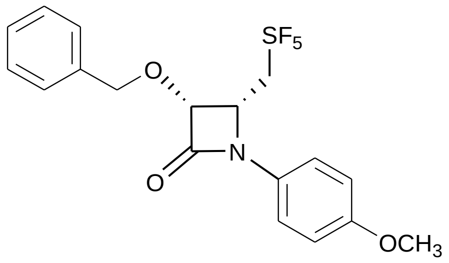

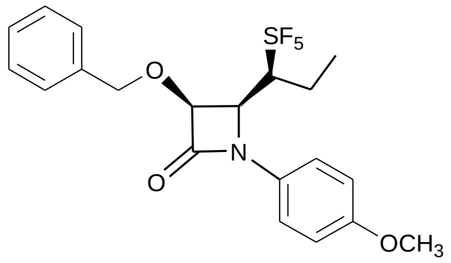

Table S1. Crystallographic data and X-ray experimental conditions for **7a** and **7c**.

	**7a**	**7c**
Empirical formula	$C_{18}H_{18}F_5NO_3S$	$C_{20}H_{22}F_5NO_3S$
Formula weight	423.39	451.45
T (K)	100(2)	100(2)
Crystal system	monoclinic	triclinic
Space group	$P2_1/c$	$P\bar{1}$
a (Å)	5.9580(11)	6.809 (7) Å
b (Å)	14.455(3)	10.145 (10) Å
c (Å)	20.381(4)	14.616 (15) Å
α (°)	90.00	93.168 (13)
β (°)	92.069(2)	95.116 (14)°
γ (°)	90.00	97.126 (13)
V (Å^3)	1754.1(6)	995.6 (18)
Z	4	2
$R1^a$, $wR2^b$ [$I > 2\sigma(I)$]	0.0322, 0.0867	0.1262, 0.2922
$R1^a$, $wR2^b$ (all data)	0.0352, 0.0898	0.2002, 0.3241
Quality-of-fit^c	1.059	1.051
Largest diff. peak/hole, e/Å^3	0.382, -0.408	1.159, -0.956

$^aR1 = \Sigma||F_o|-|F_c||/\Sigma|F_o|$

$^bwR2 = [\Sigma[w(F_o^2-F_c^2)^2]/\Sigma[w(F_o^2)^2]]^{½}$

^{c}Quality-of-fit = $[\Sigma[w(F_o^2-F_c^2)^2]/(N_{obs}-N_{params})]^{½}$, based on all data.

NMR Spectra

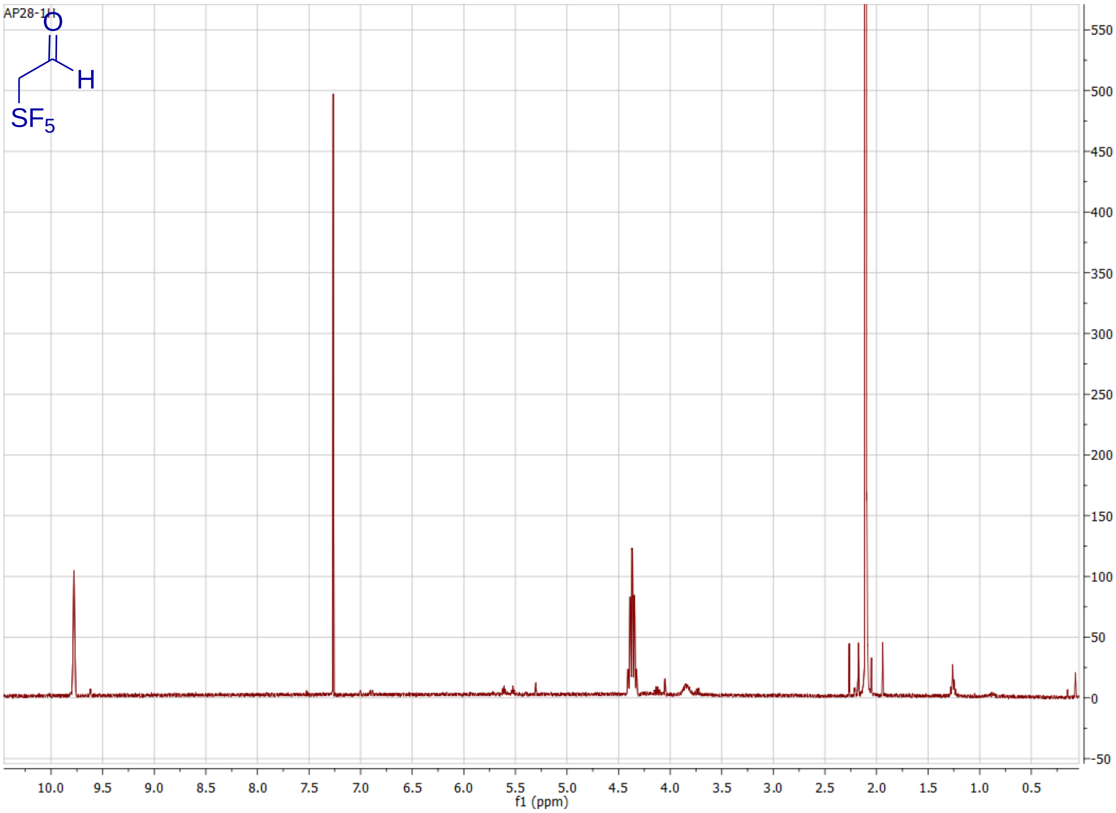

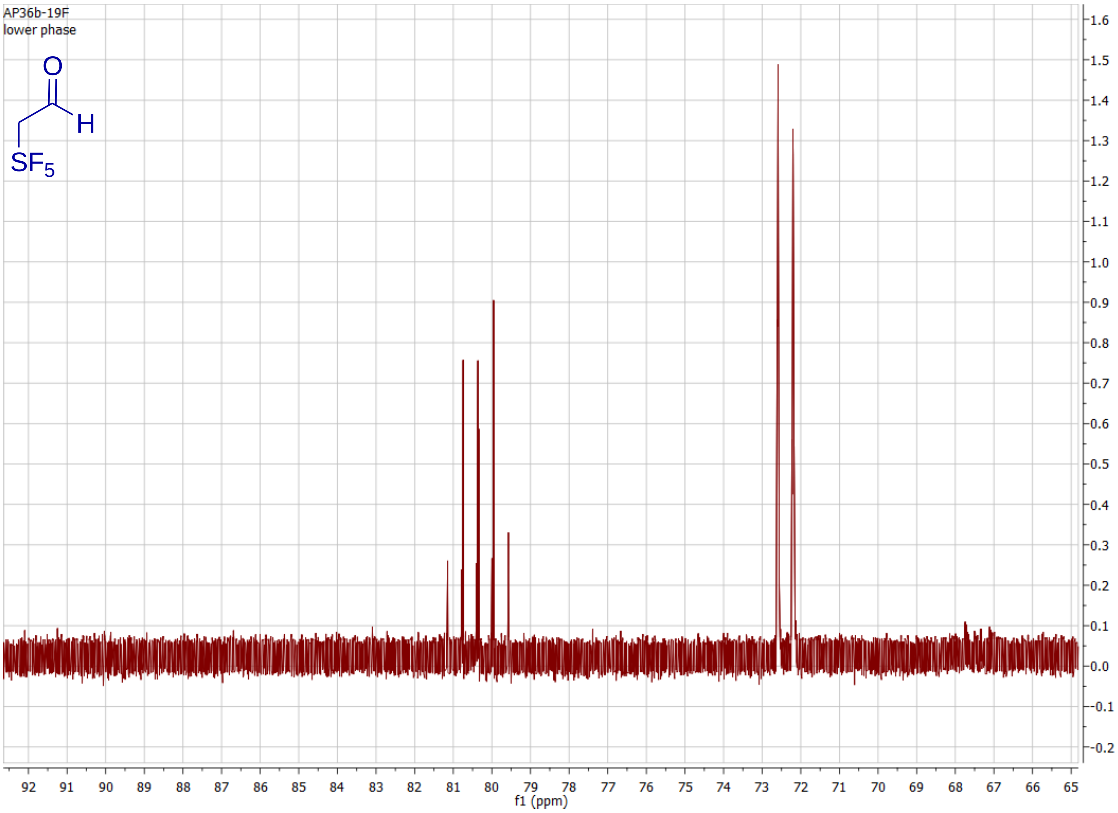

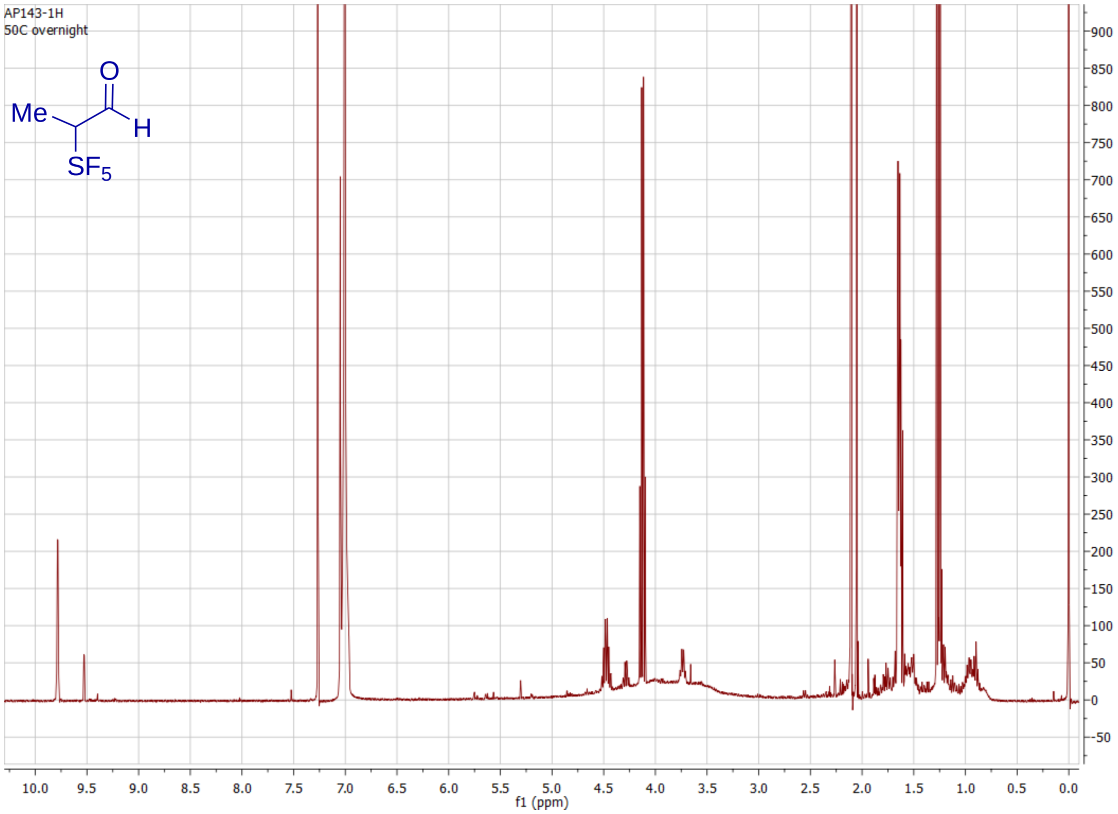
AP143-1H
50C overnight
O
Me
H
SF5
f1 (ppm)

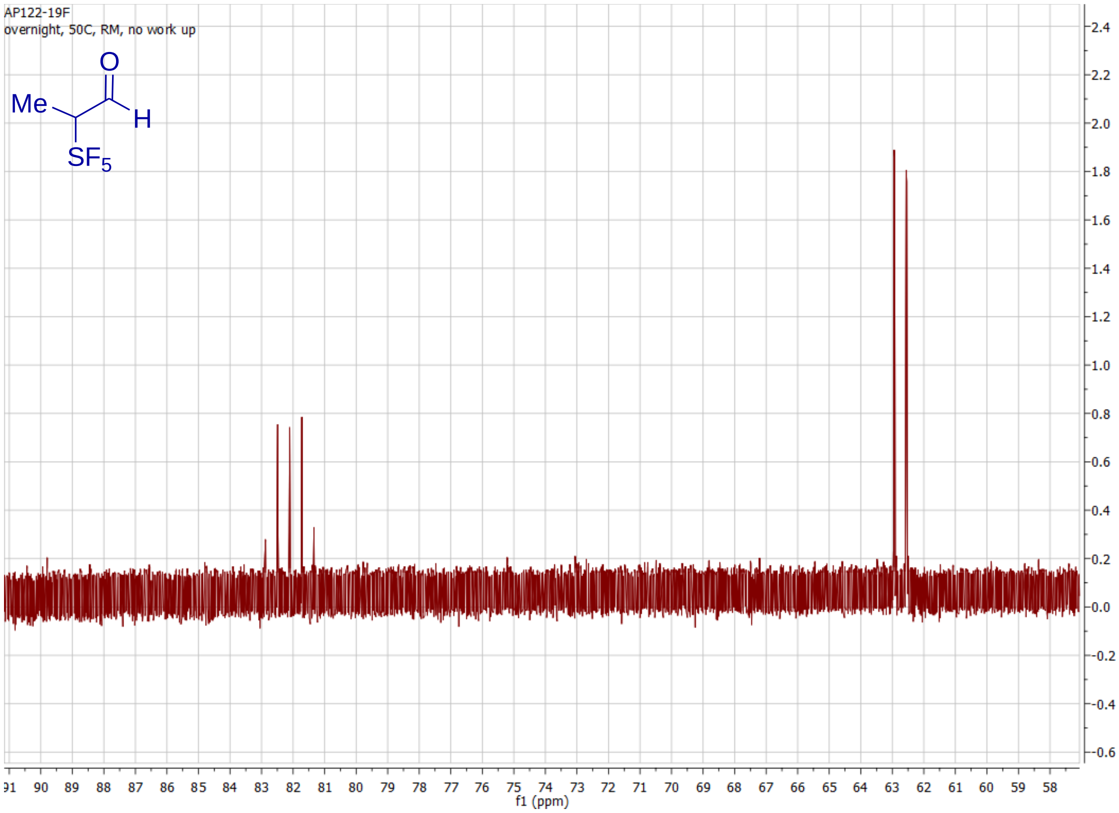
AP122-19F
overnight, 50C, RM, no work up
O
Me
H
SF5
f1 (ppm)

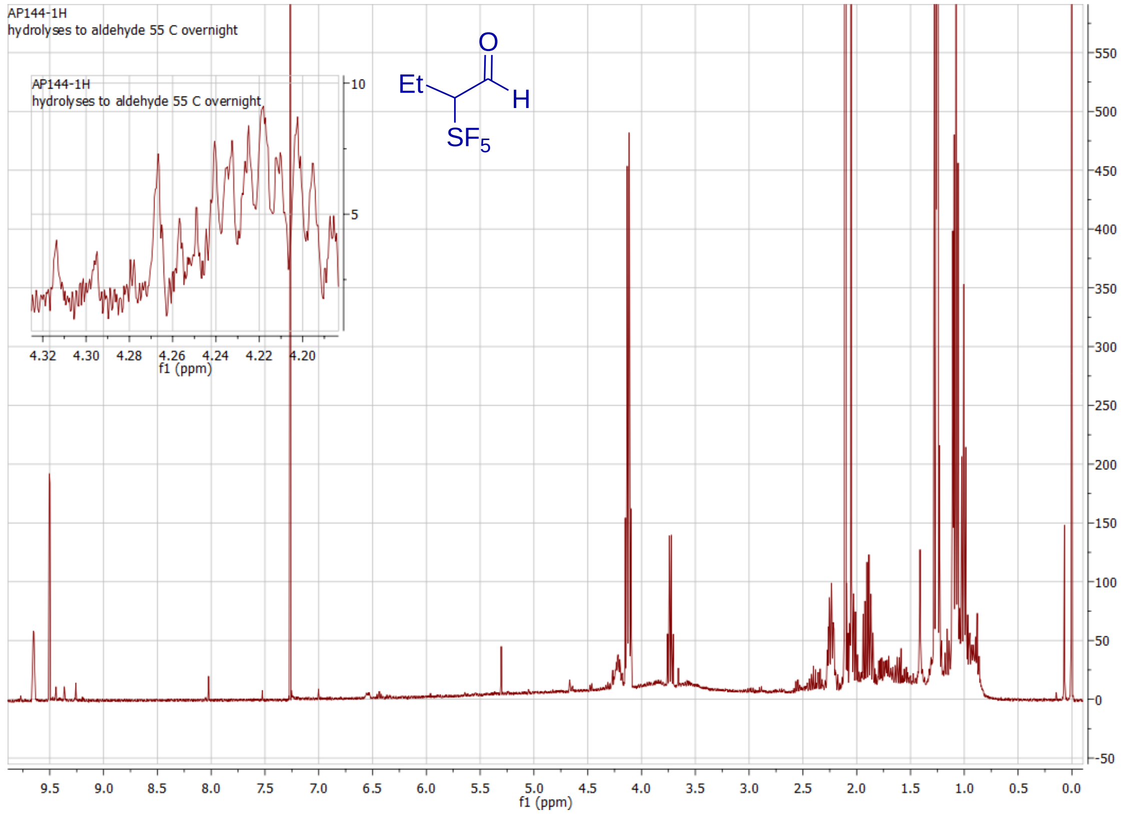
AP144-1H
hydrolyses to aldehyde 55 C overnight
AP144-1H
hydrolyses to aldehyde 55 C overnight
Et
O
H
SF5
f1 (ppm)
f1 (ppm)

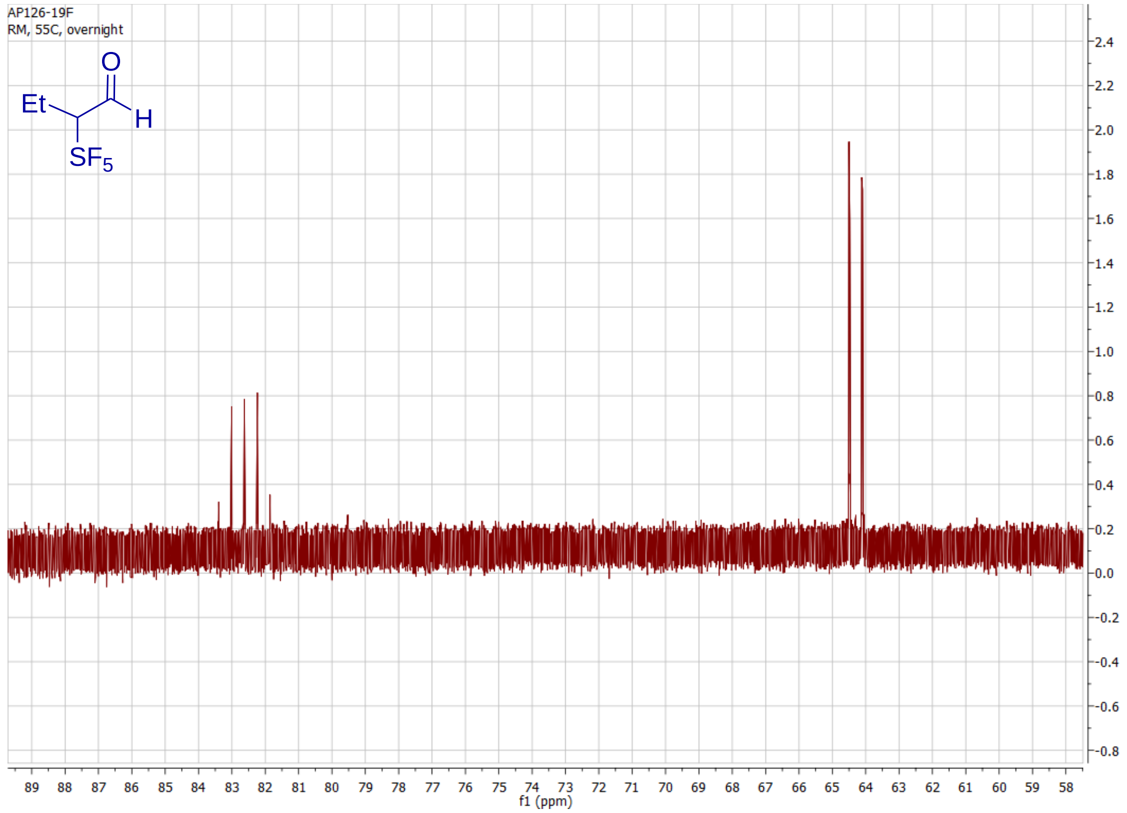
AP126-19F
RM, 55C, overnight
Et
O
H
SF5
f1 (ppm)

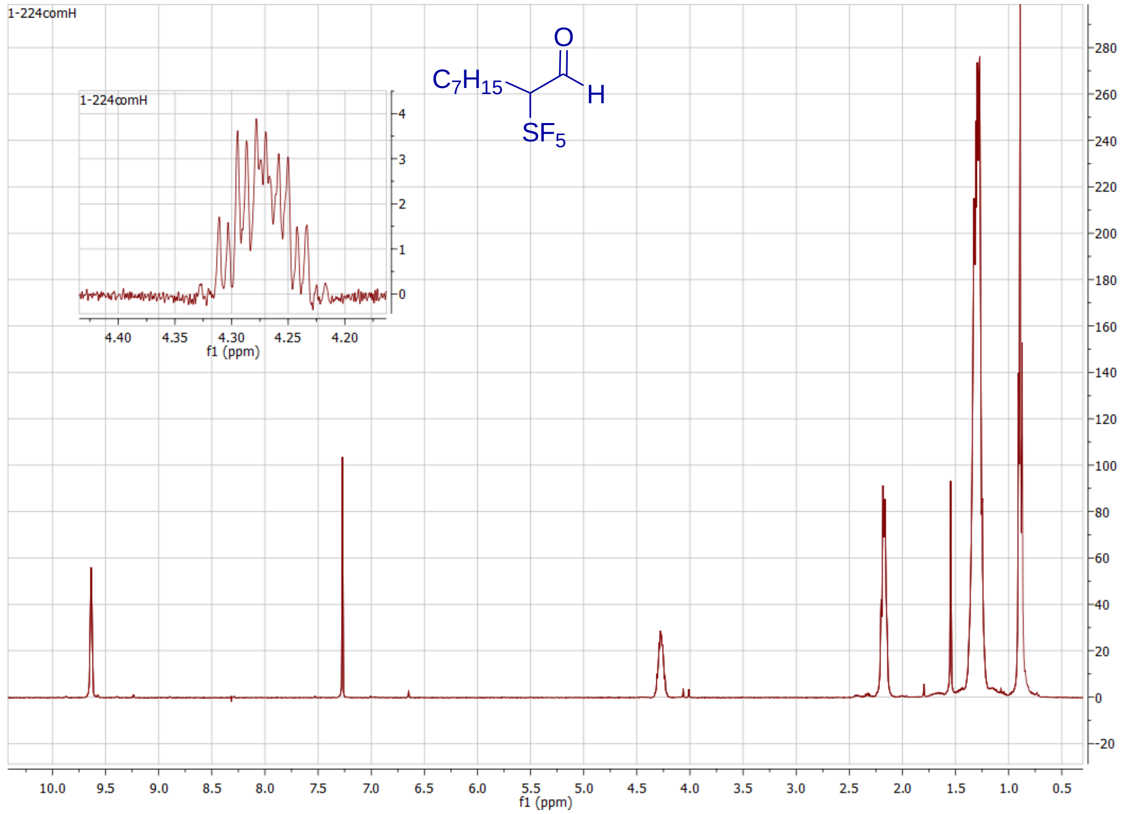
1-224comH
1-224comH
f1 (ppm)
C7H15
O
H
SF5

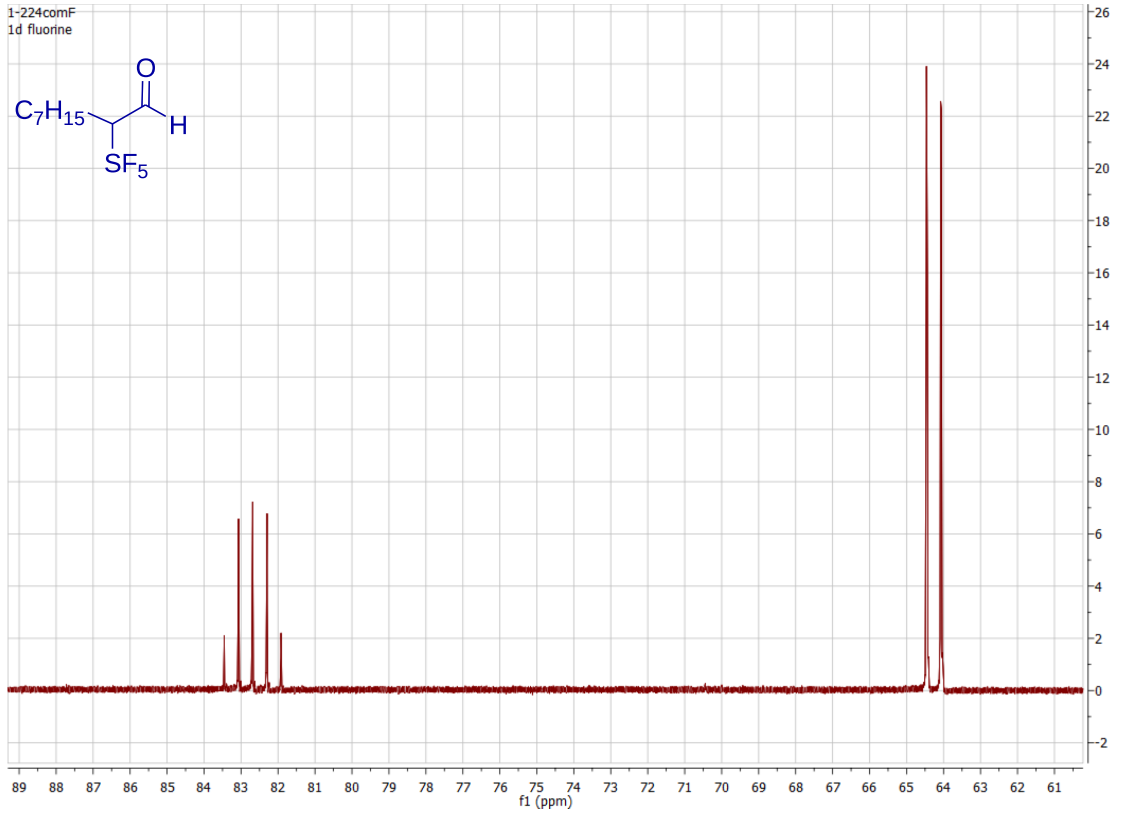
1-224comF
1d fluorine
C7H15
O
H
SF5
f1 (ppm)

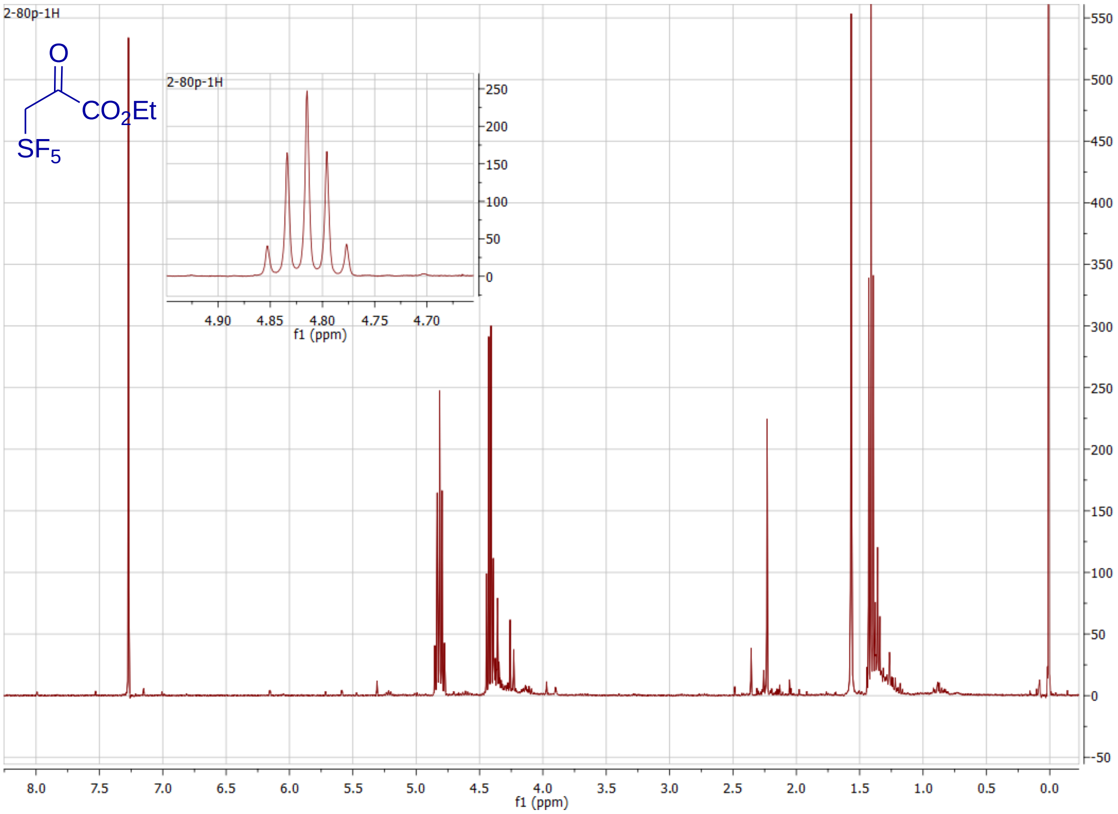
2-80p-1H
SF5
CO2Et
O
f1 (ppm)

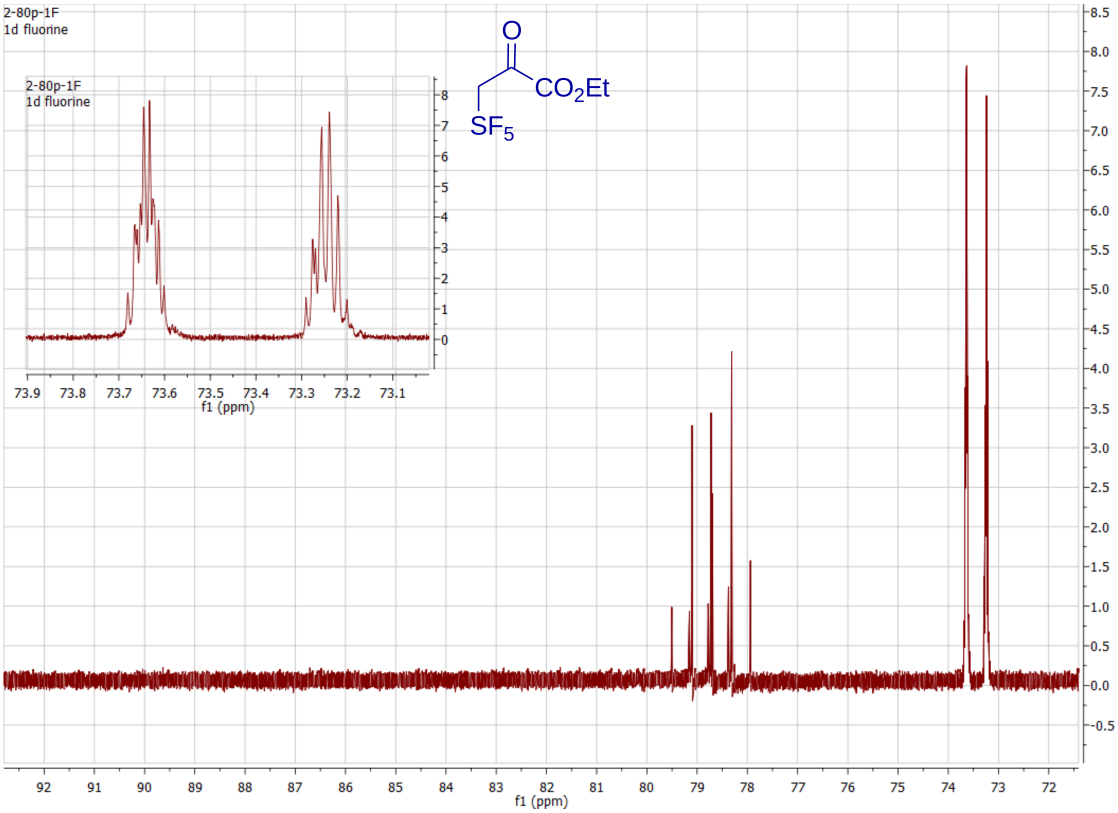
2-80p-1F
1d fluorine
SF5
CO2Et
O
f1 (ppm)

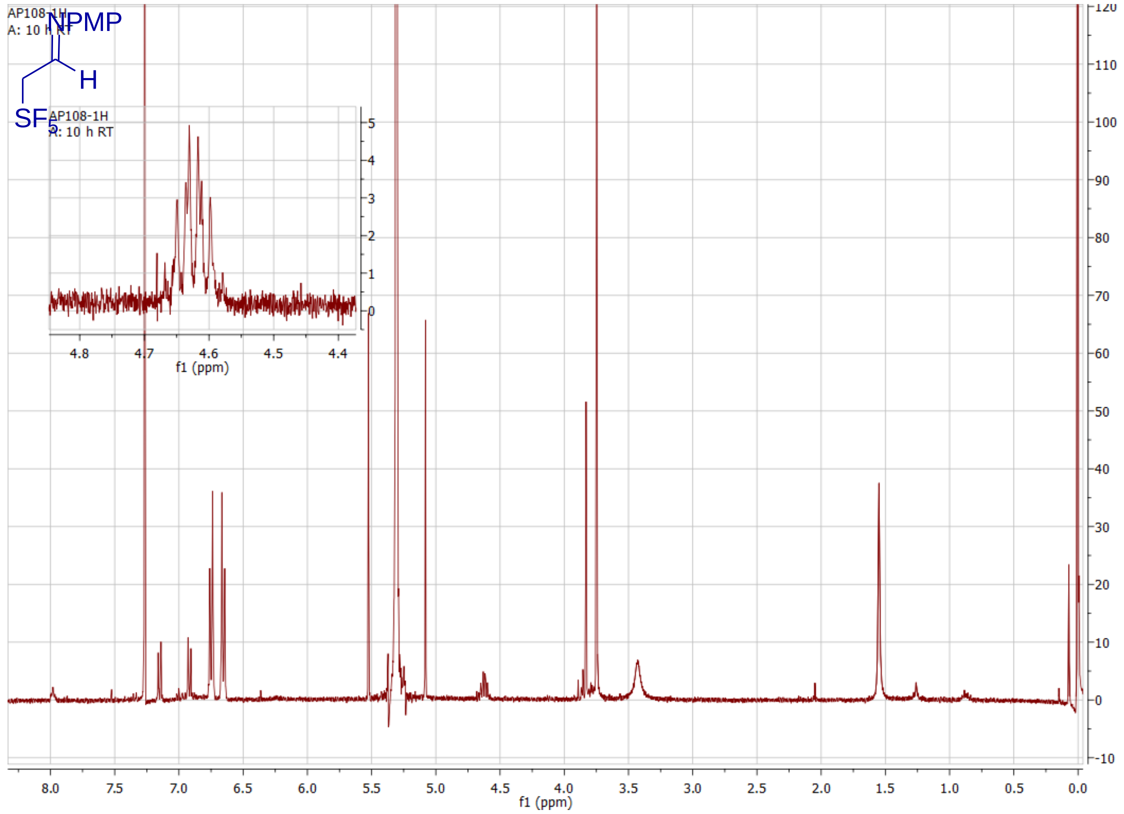
AP108-1H
A: 10 h RT
NPMP
H
SF5
f1 (ppm)

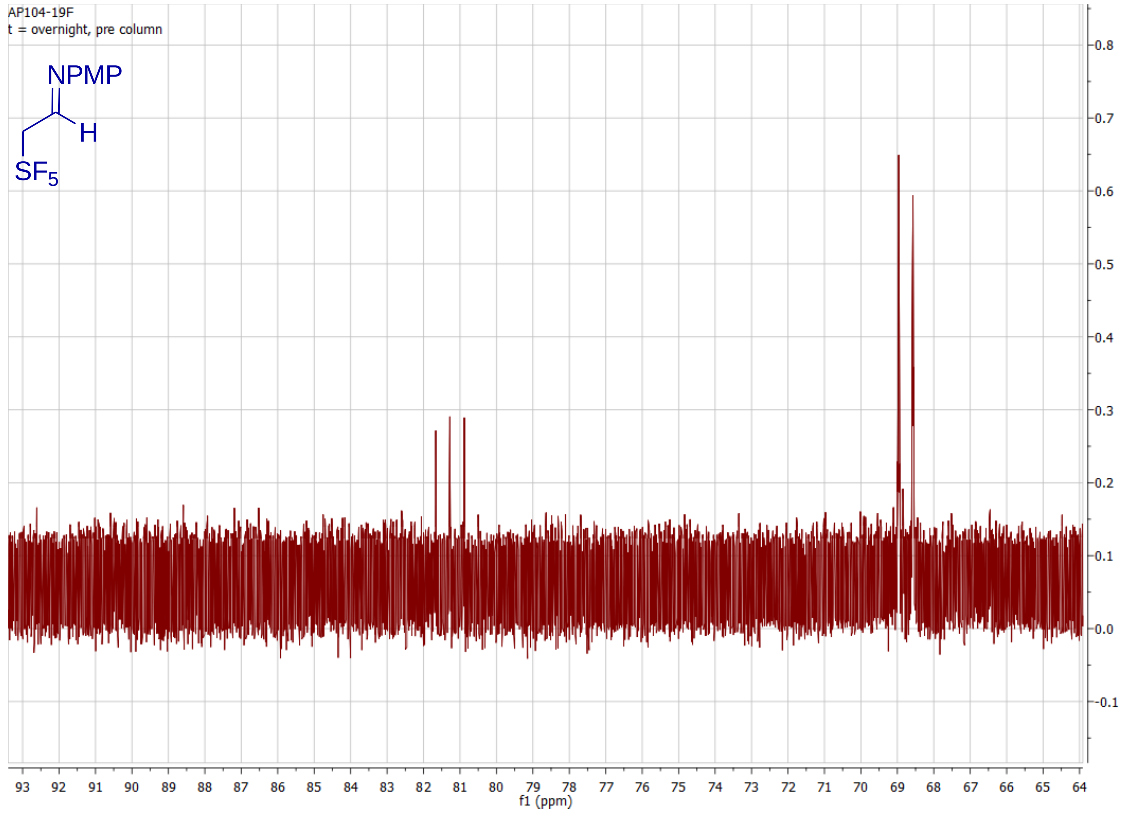
AP104-19F
t = overnight, pre column
NPMP
H
SF5
f1 (ppm)

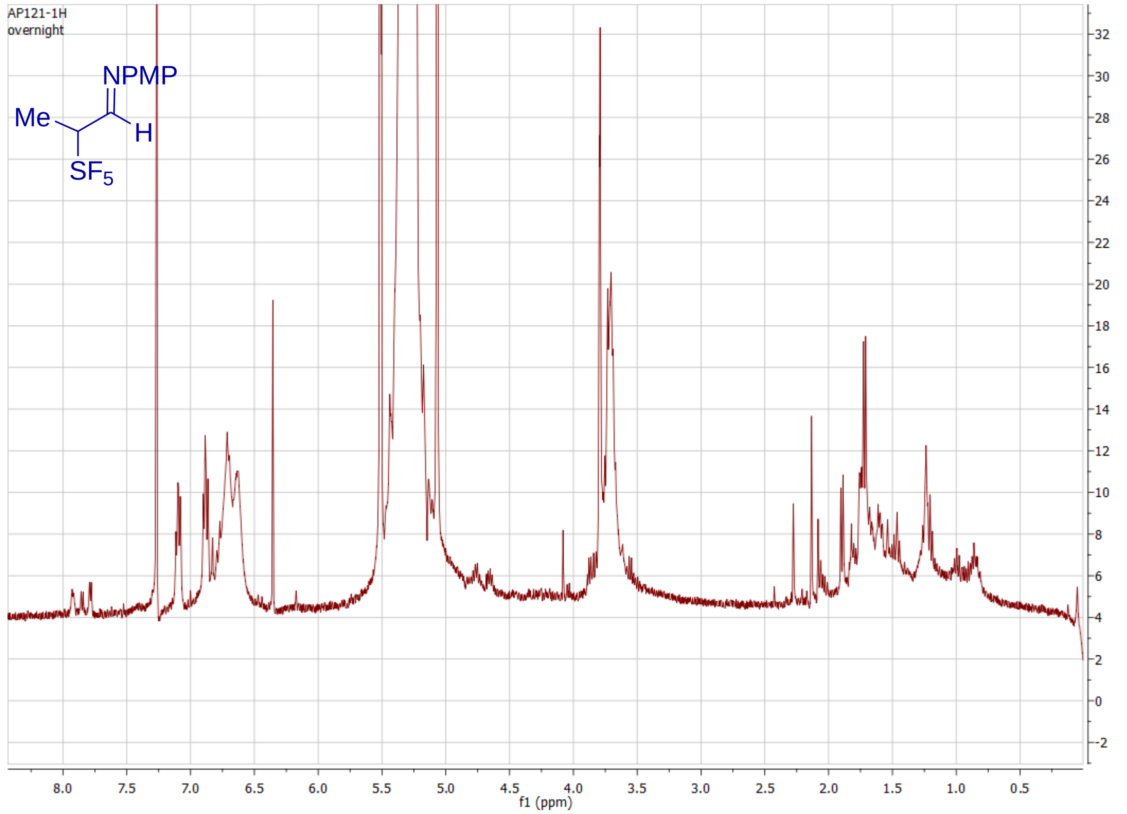
AP121-1H
overnight
NPMP
Me
H
SF5
f1 (ppm)

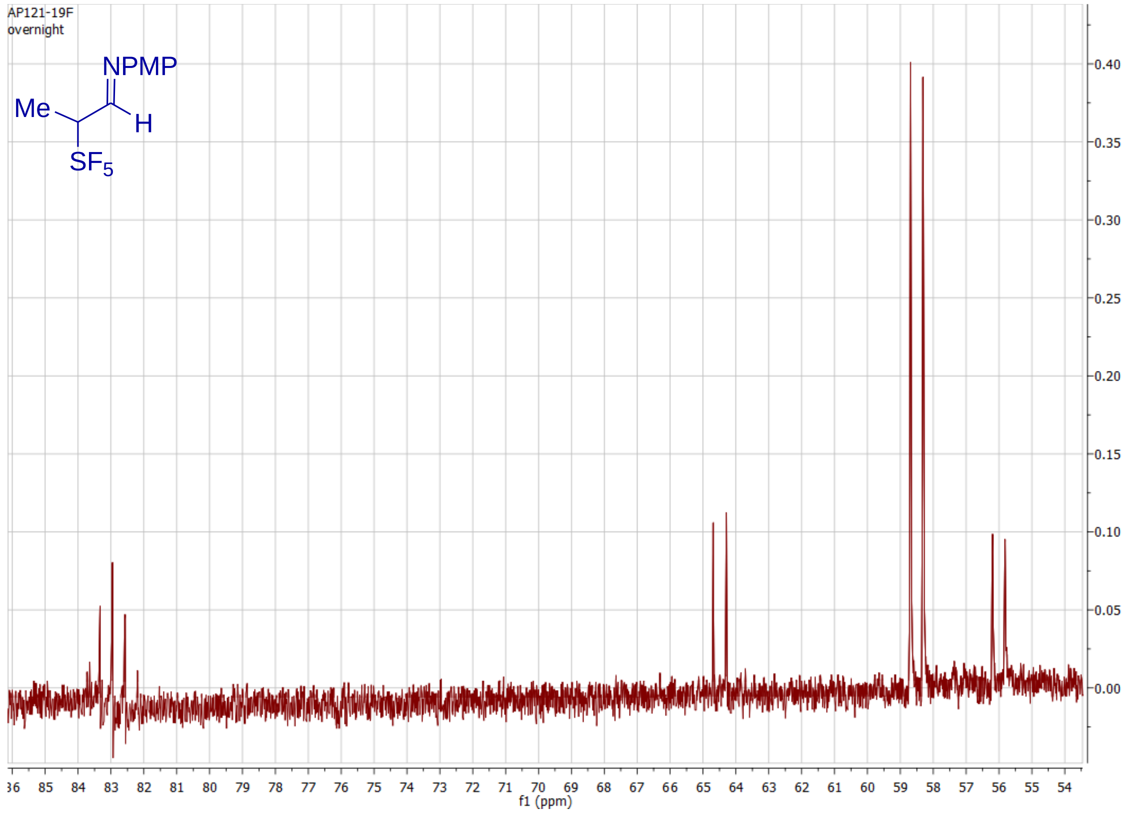
AP121-19F
overnight
NPMP
Me
H
SF5
f1 (ppm)

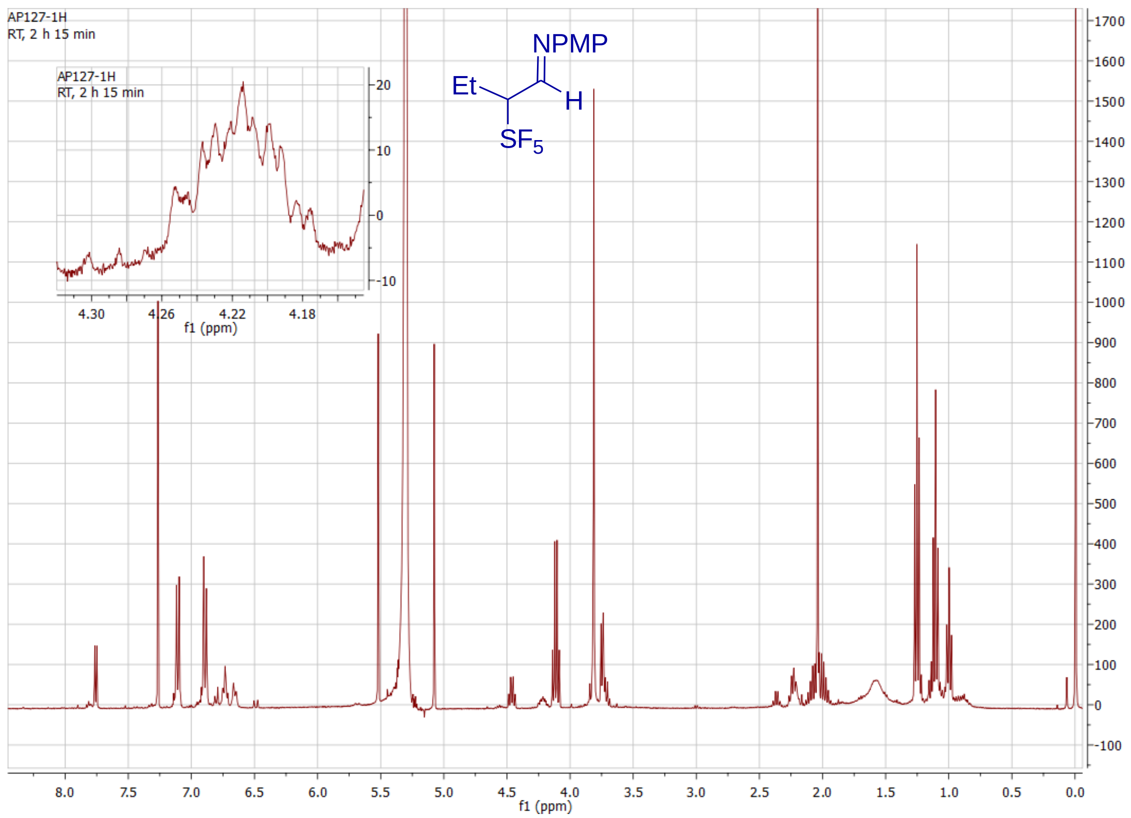
AP127-1H
RT, 2 h 15 min
NPMP
Et
H
SF5
f1 (ppm)

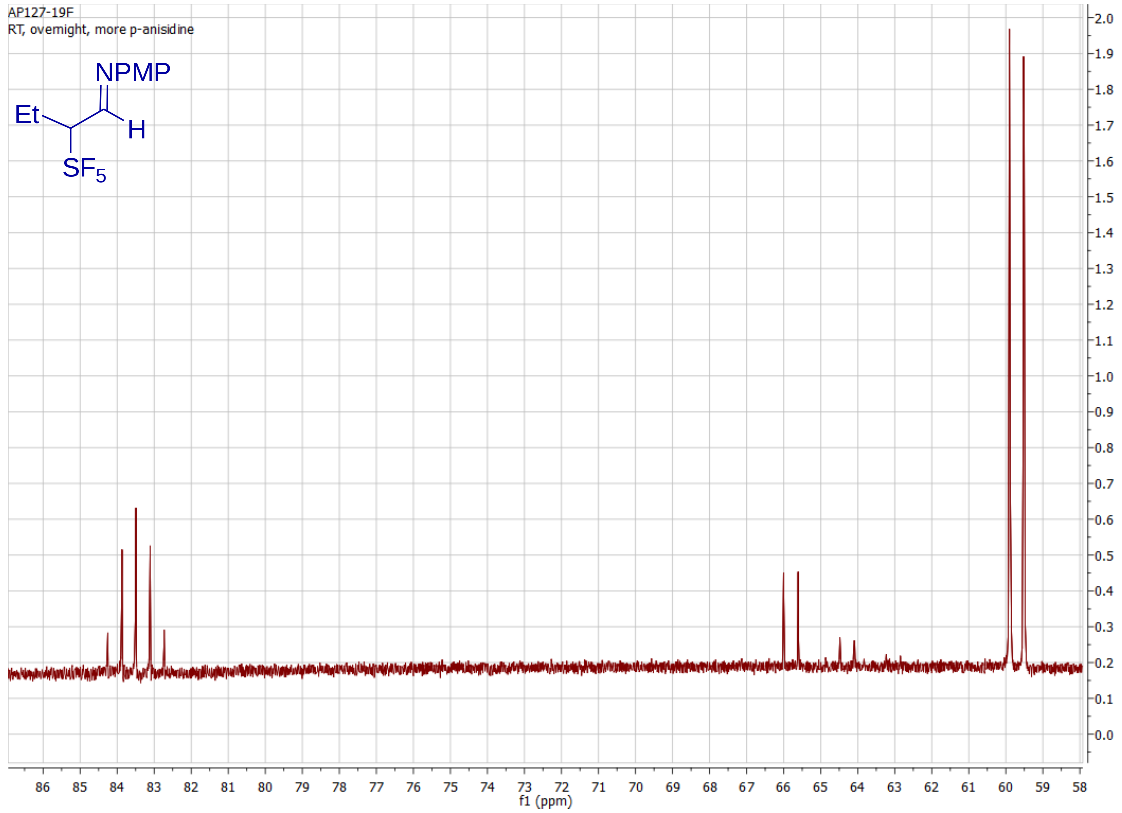
AP127-19F
RT, overnight, more p-anisidine
NPMP
Et
H
SF5
f1 (ppm)

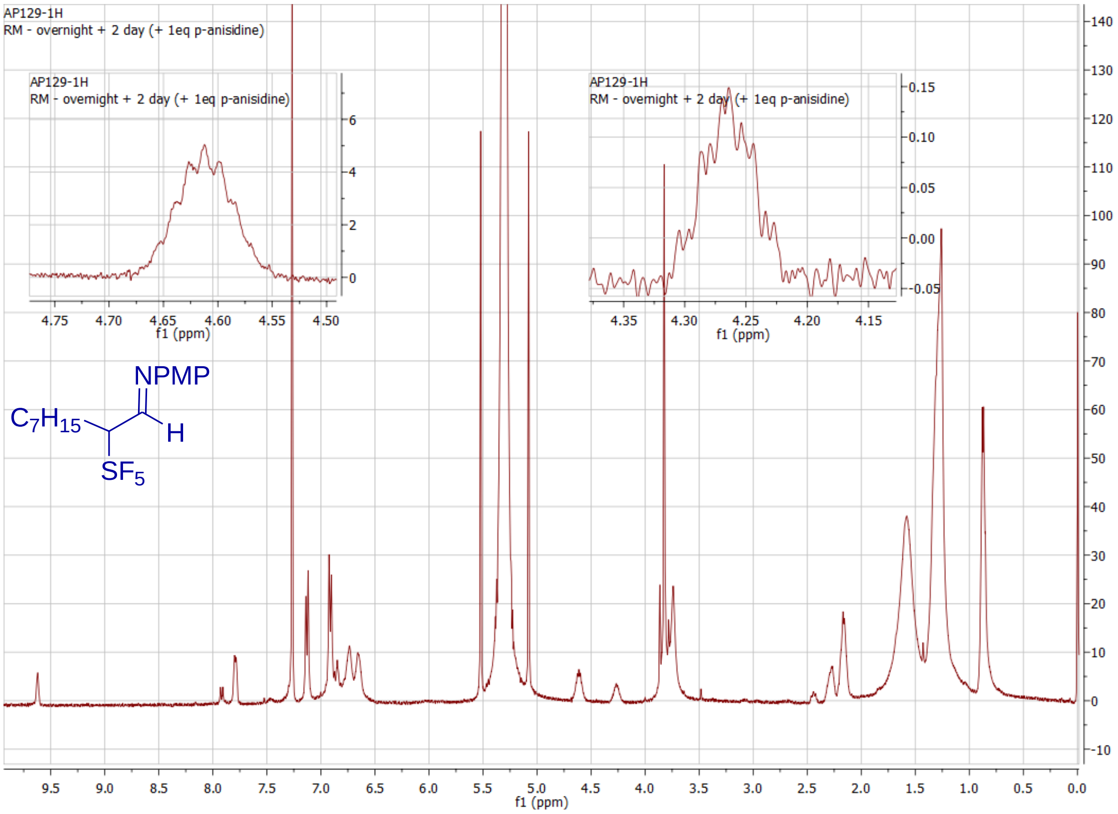
AP129-1H
RM - overnight + 2 day (+ 1eq p-anisidine)
AP129-1H
RM - overnight + 2 day (+ 1eq p-anisidine)
AP129-1H
RM - overnight + 2 day (+ 1eq p-anisidine)
f1 (ppm)
f1 (ppm)
NPMP
C_7H_{15}
H
SF_5
f1 (ppm)

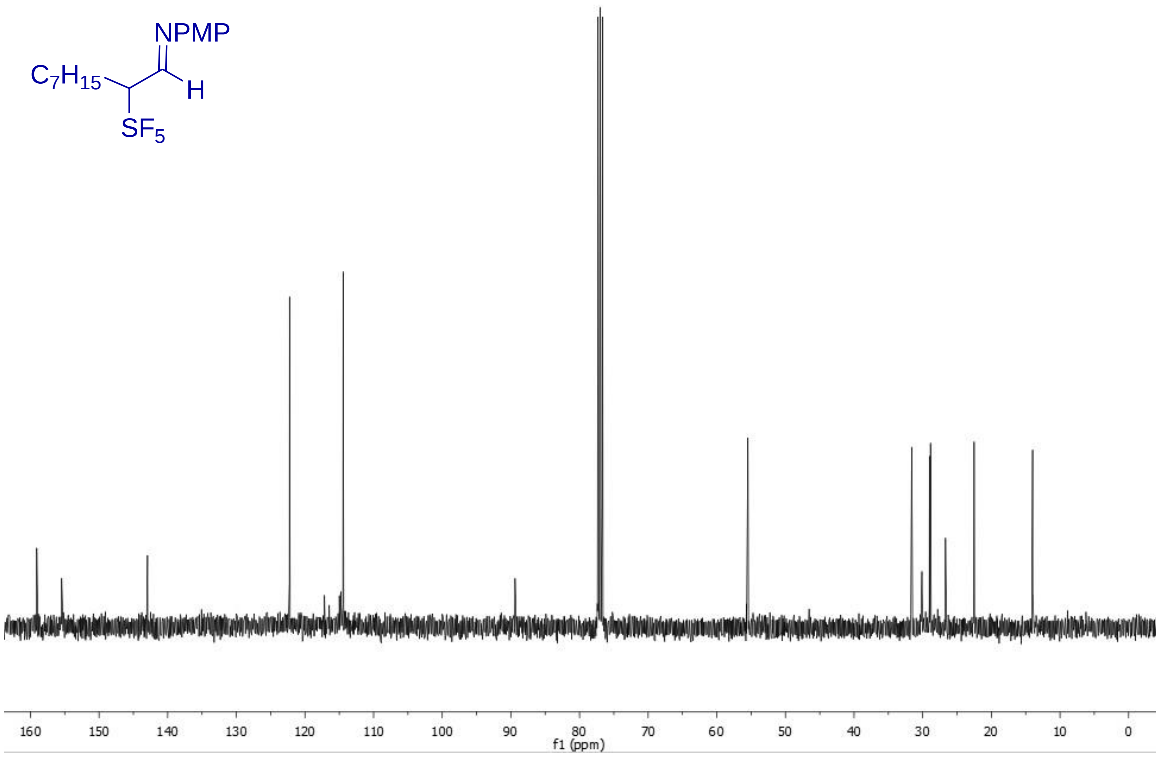
NPMP
C_7H_{15}
H
SF_5
f1 (ppm)

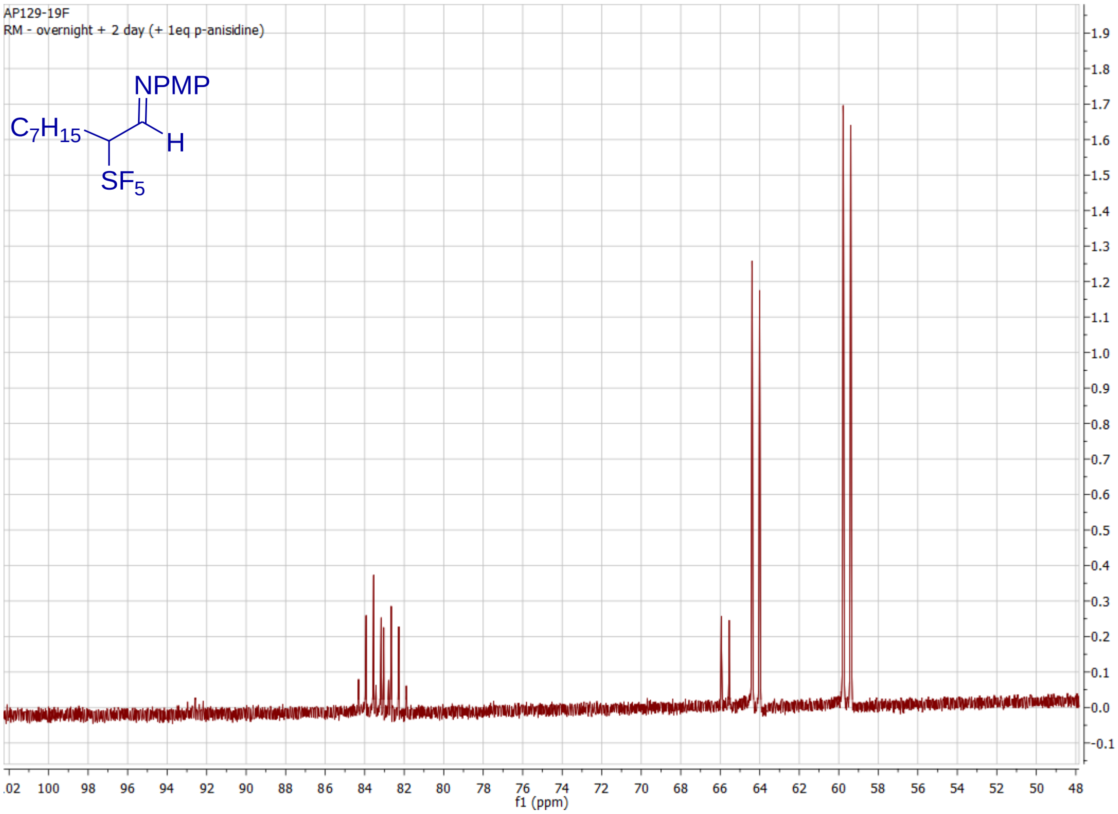

AP129-19F
RM - overnight + 2 day (+ 1eq p-anisidine)
NPMP
C_7H_{15}
H
SF_5
f1 (ppm)

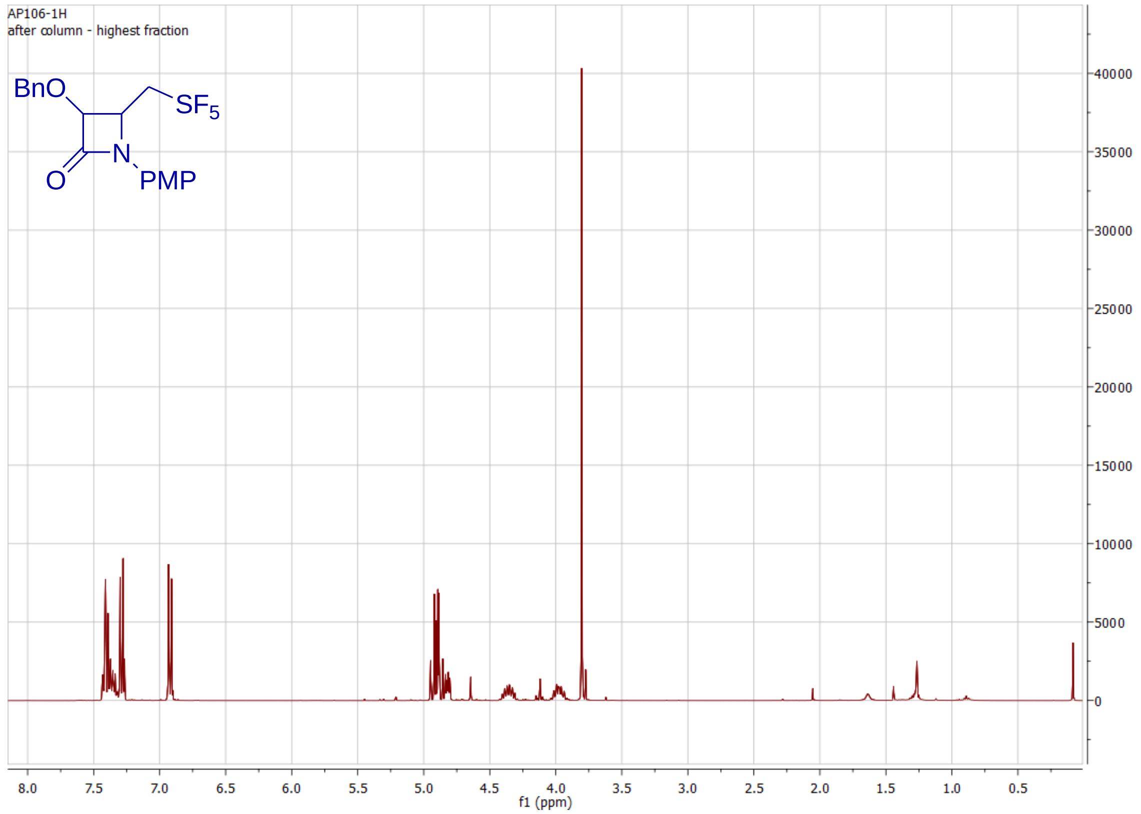
AP106-1H
after column - highest fraction
BnO
SF5
N
O
PMP
f1 (ppm)

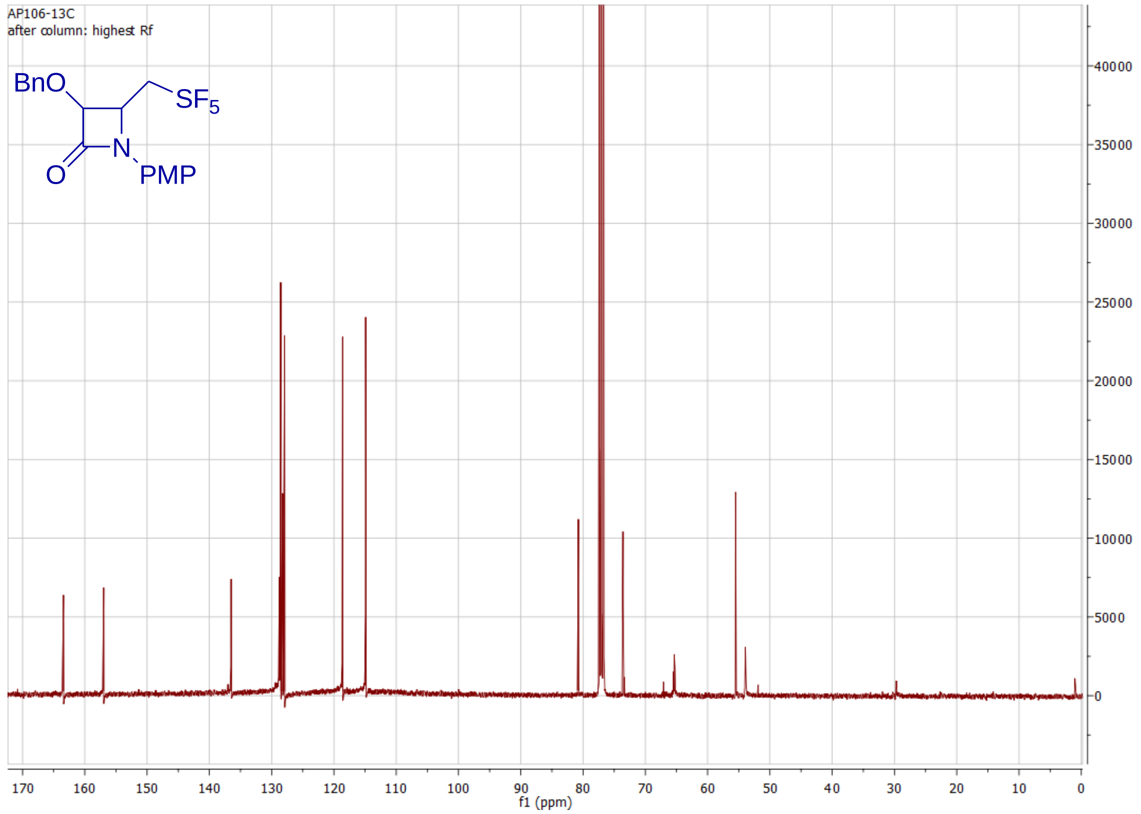
AP106-13C
after column: highest Rf
BnO
SF5
N
O
PMP
f1 (ppm)

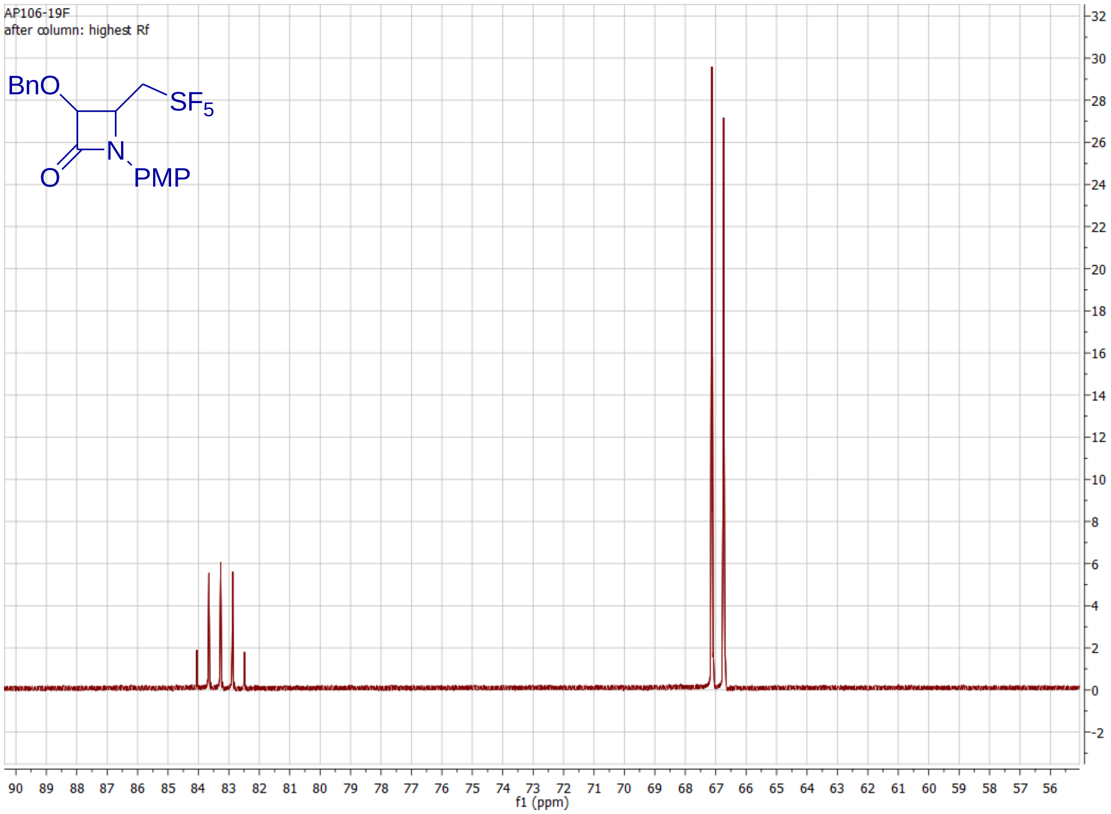

AP106-19F
after column: highest Rf
BnO
SF5
N
O
PMP
90 89 88 87 86 85 84 83 82 81 80 79 78 77 76 75 74 73 72 71 70 69 68 67 66 65 64 63 62 61 60 59 58 57 56
f1 (ppm)
32 30 28 26 24 22 20 18 16 14 12 10 8 6 4 2 0 -2

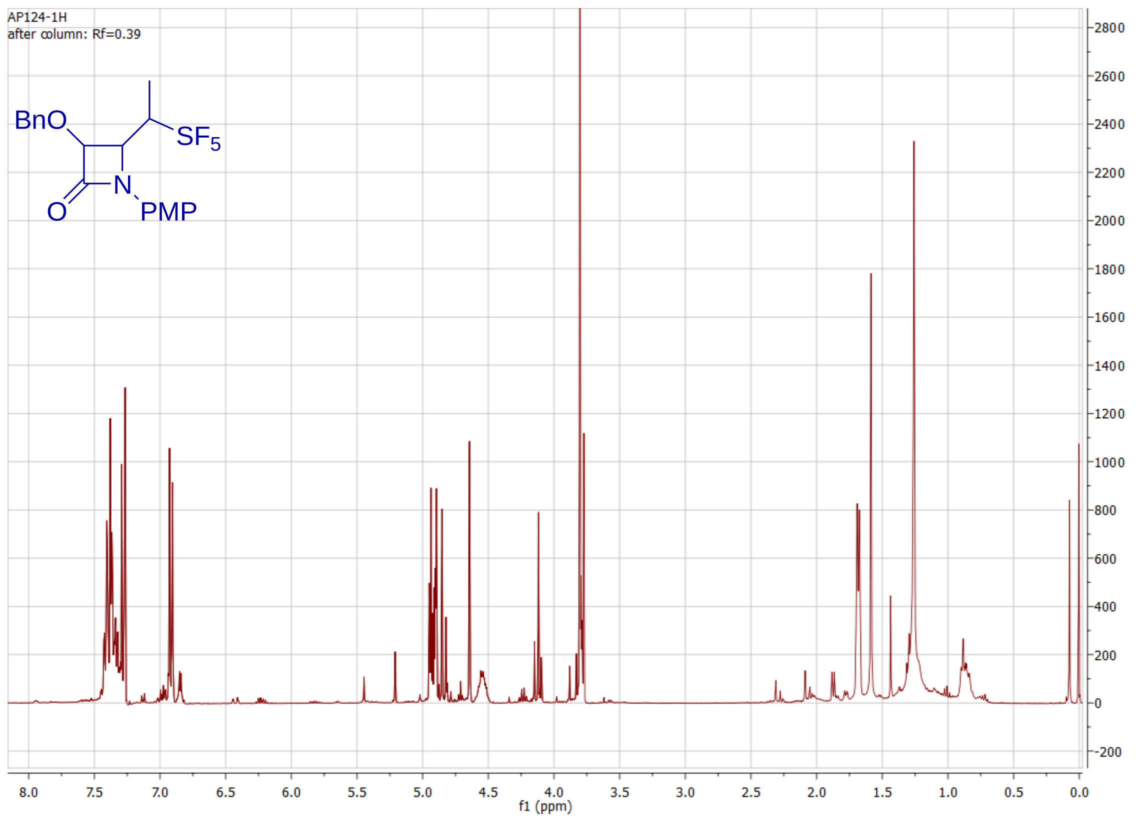
AP124-1H
after column: Rf=0.39
BnO
SF5
N
O
PMP
f1 (ppm)

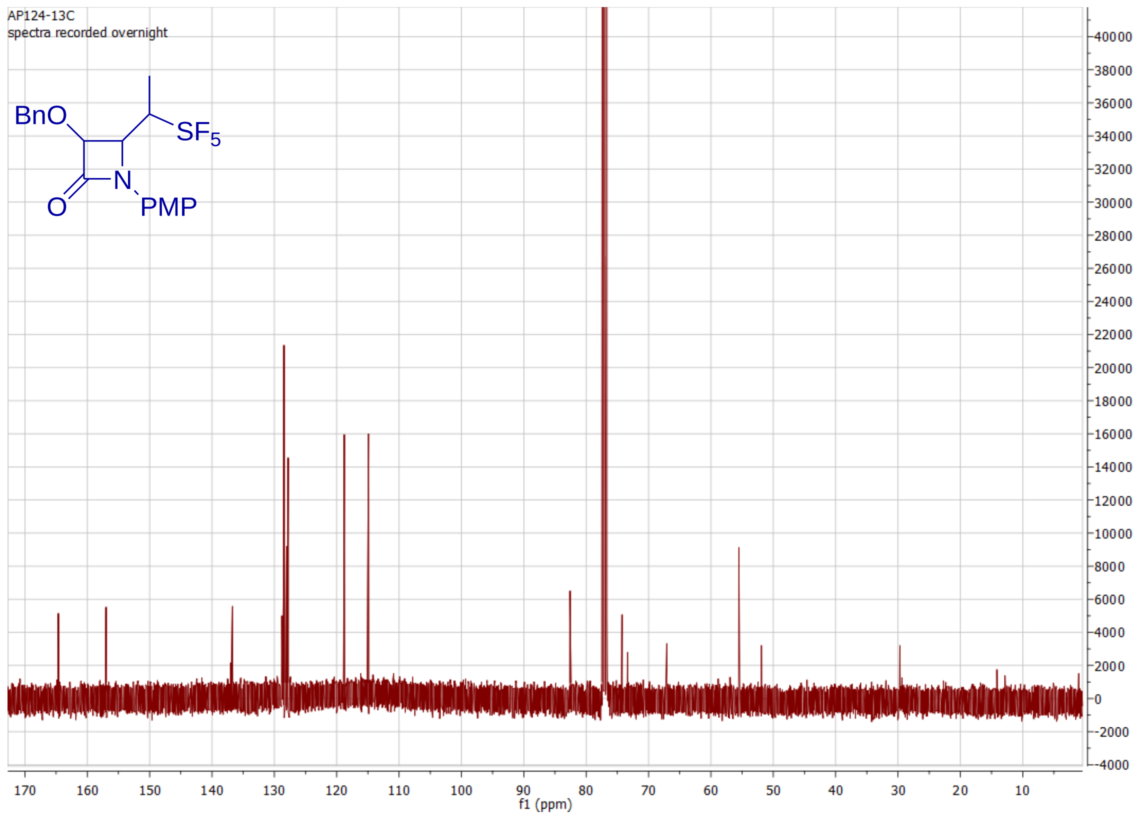
AP124-13C
spectra recorded overnight
BnO
SF5
N
O
PMP
f1 (ppm)

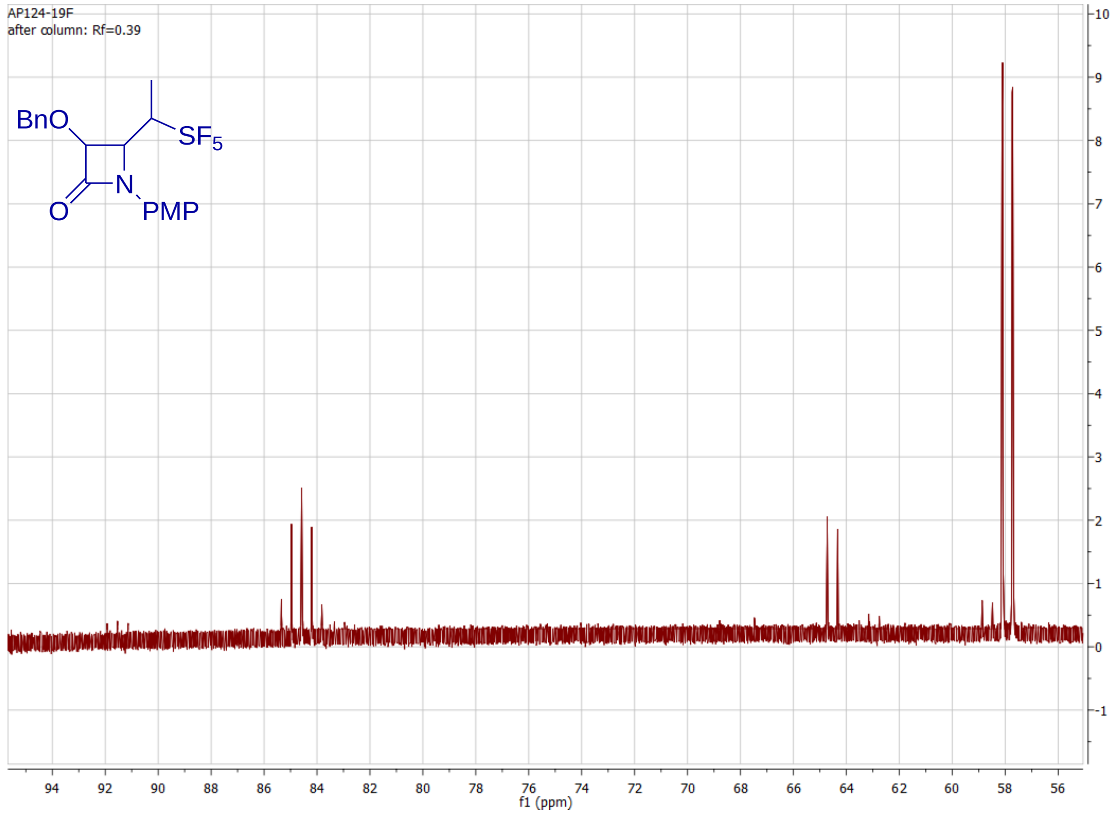

AP124-19F
after column: Rf=0.39
BnO
SF5
N
O
PMP
f1 (ppm)

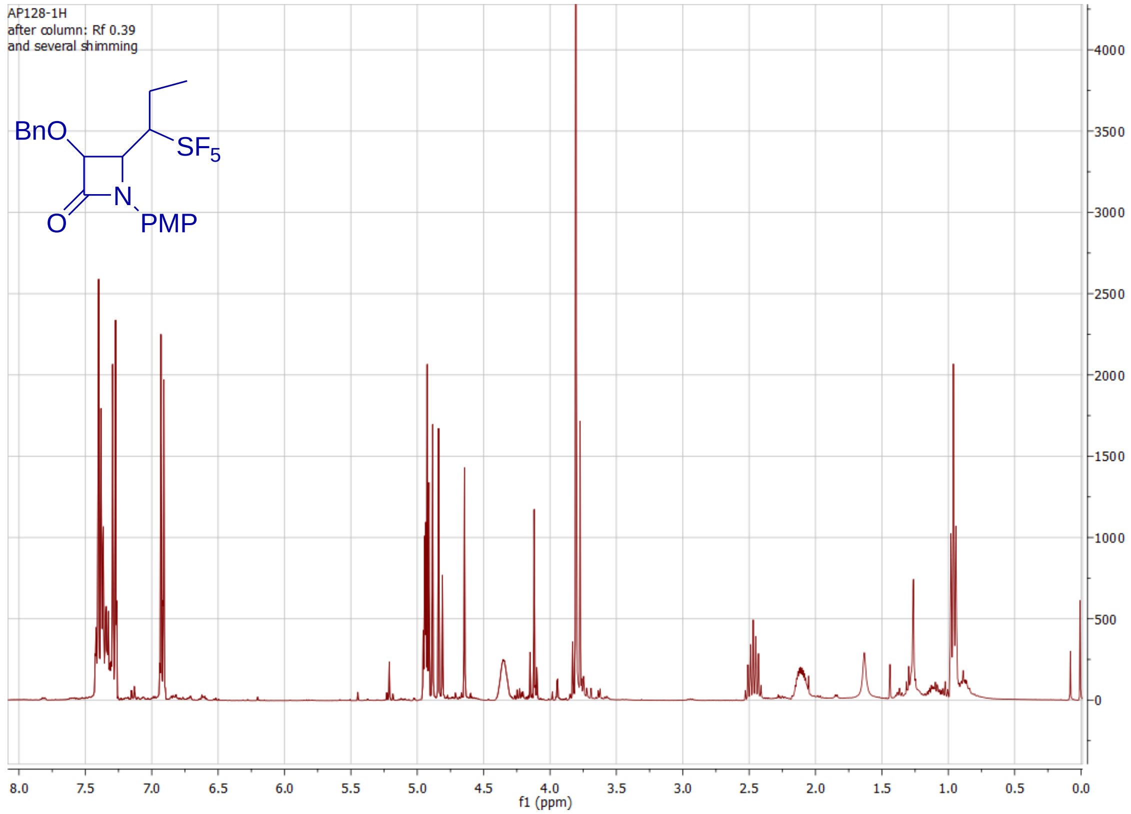
AP128-1H
after column: Rf 0.39
and several shimming
BnO
SF5
O
N
PMP
f1 (ppm)

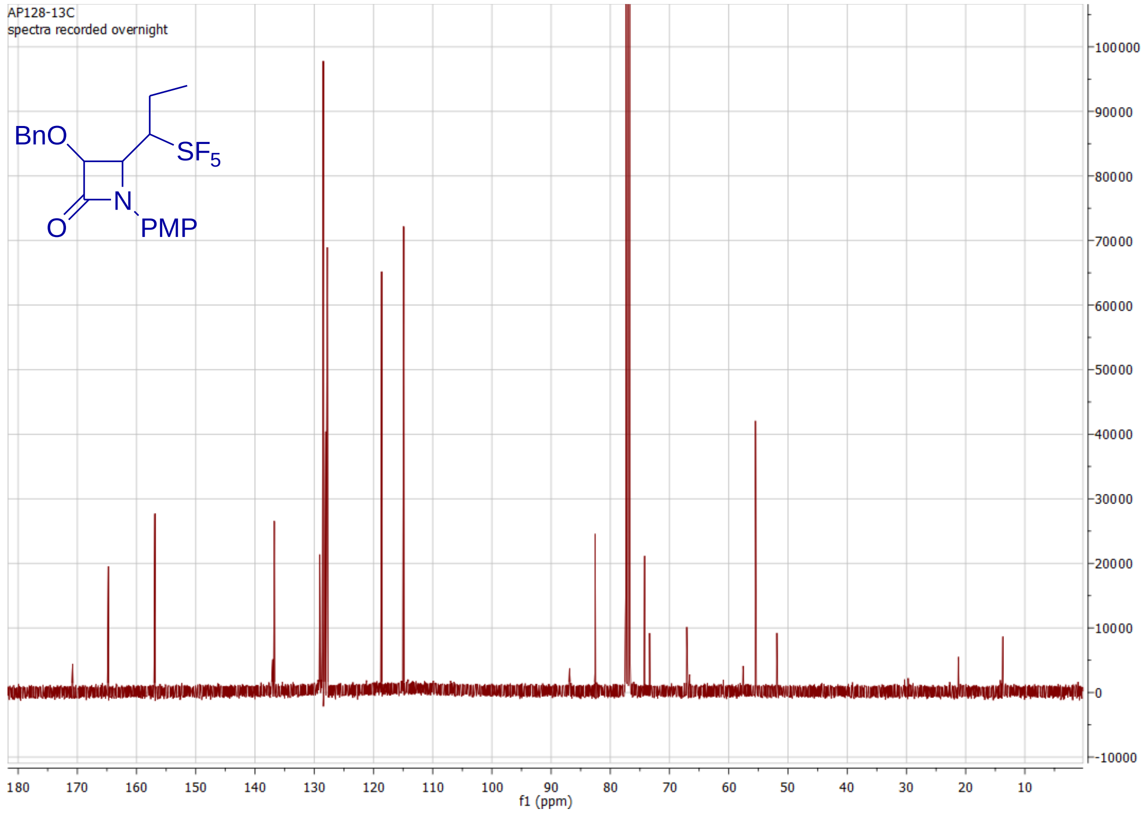
AP128-13C
spectra recorded overnight
BnO
SF5
O
N
PMP
f1 (ppm)

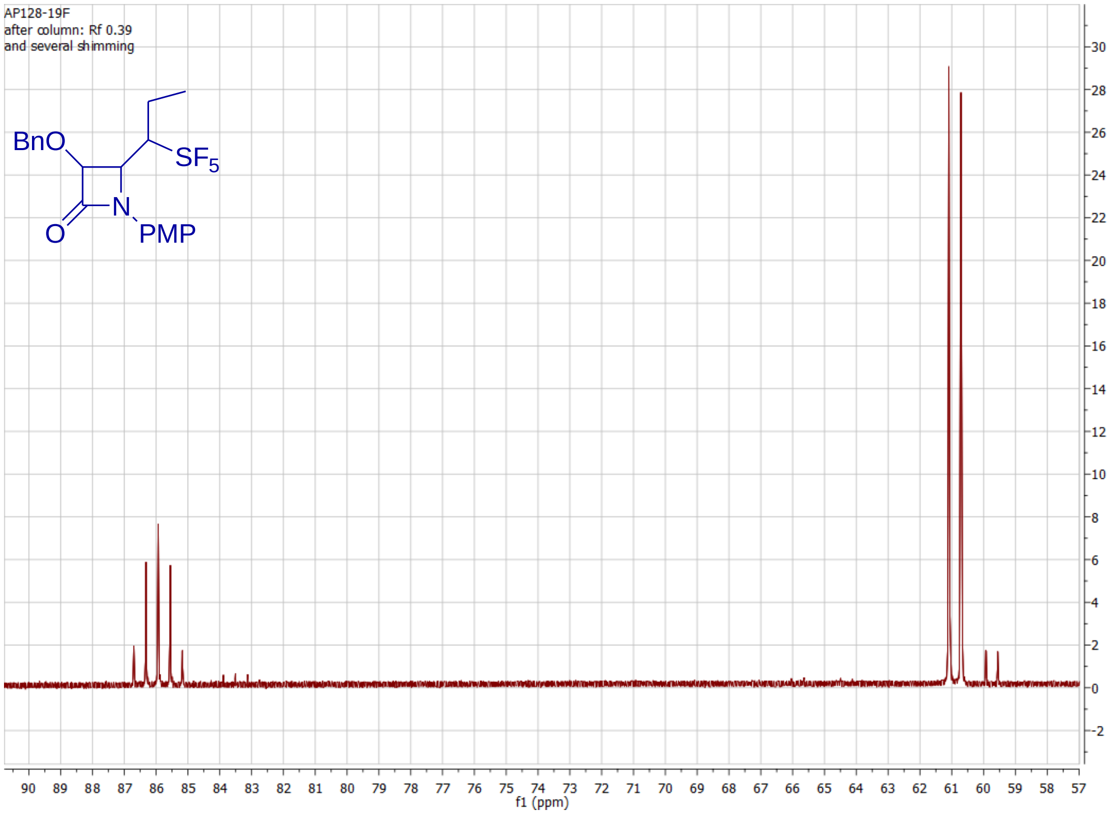
AP128-19F
after column: Rf 0.39
and several shimming
BnO
SF5
N
O
PMP
f1 (ppm)

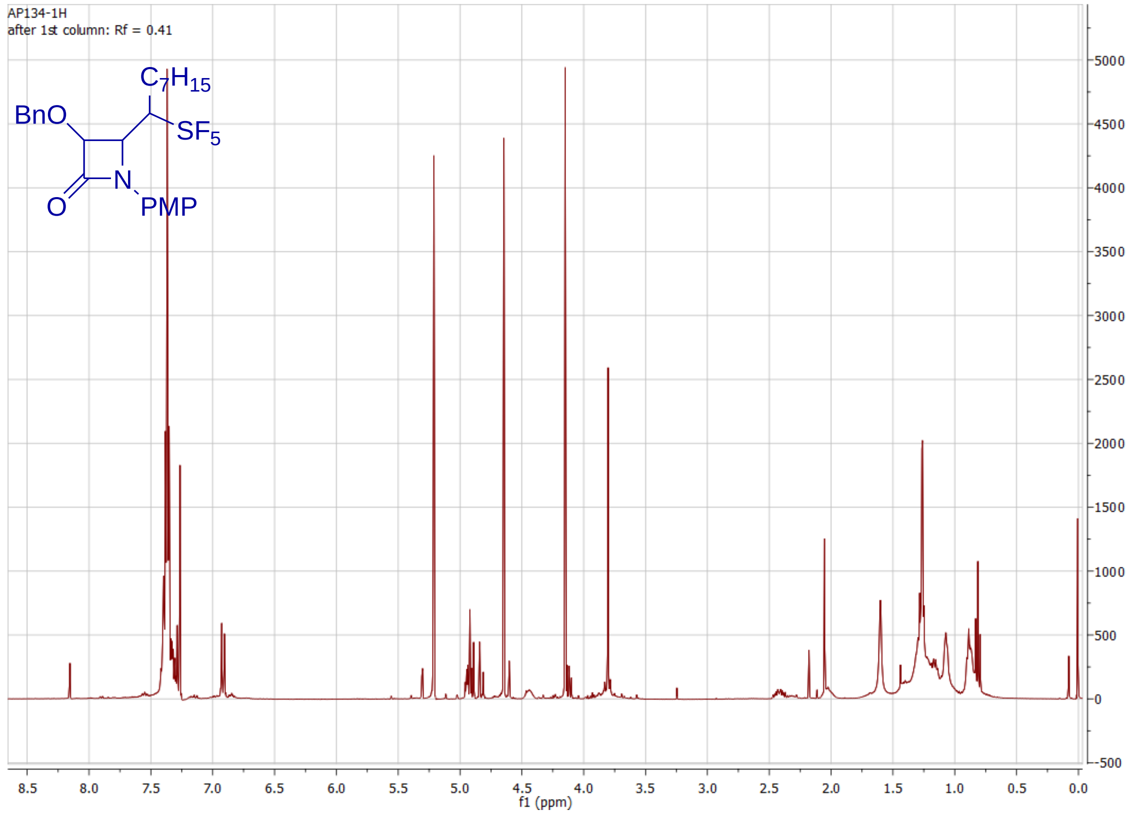
AP134-1H
after 1st column: Rf = 0.41
BnO
C7H15
SF5
N
O
PMP
f1 (ppm)

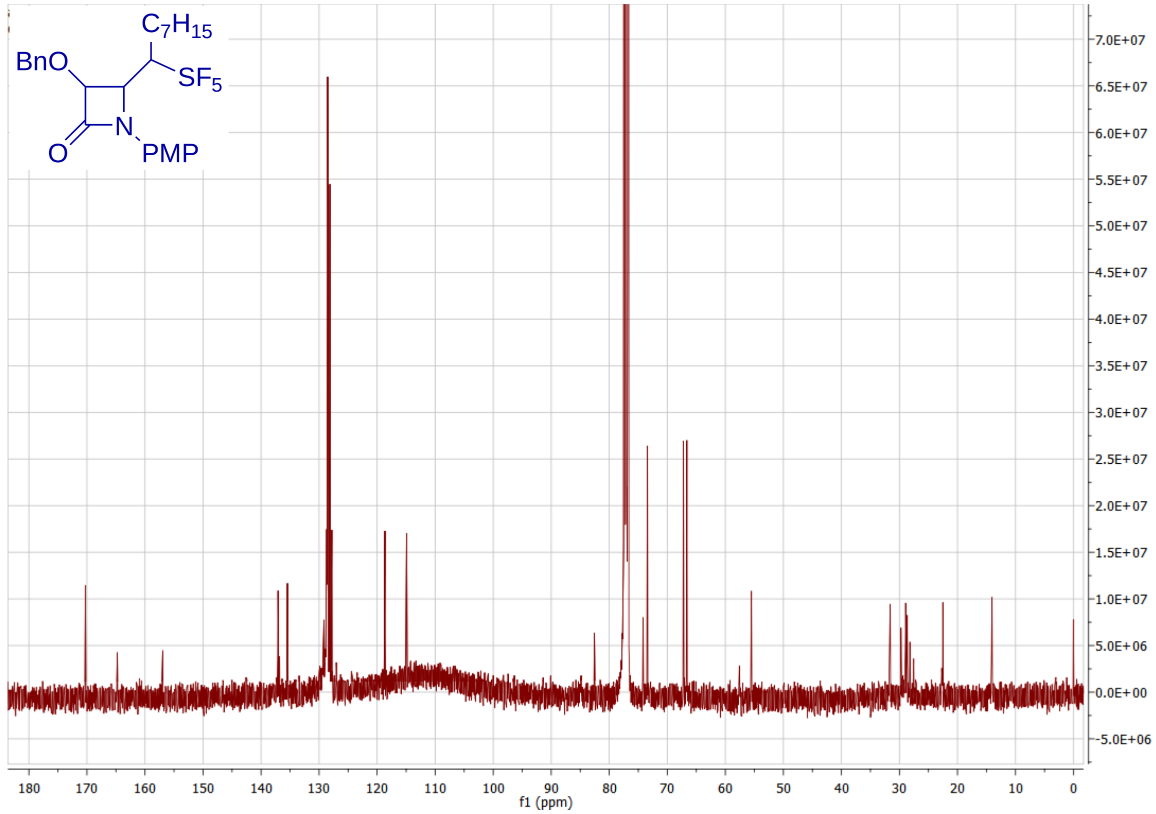
BnO
C7H15
SF5
N
O
PMP
f1 (ppm)

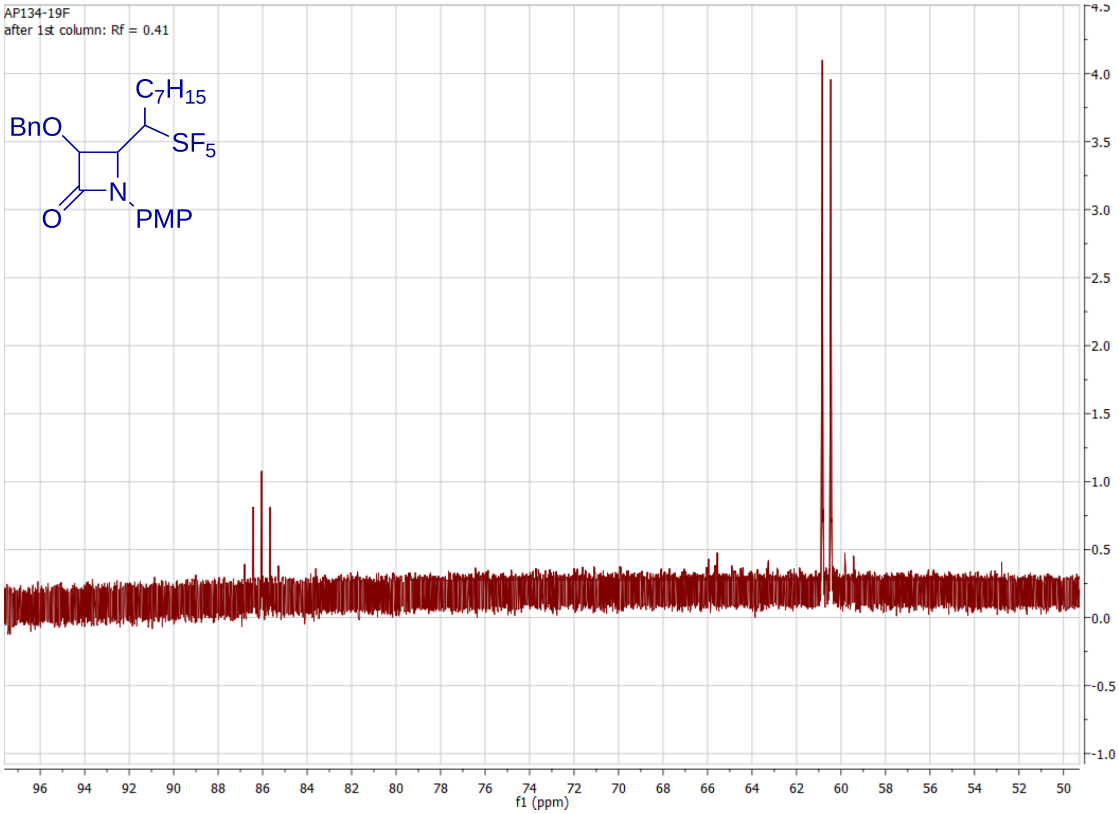
AP134-19F
after 1st column: Rf = 0.41
BnO
C7H15
SF5
N
O
PMP
f1 (ppm)

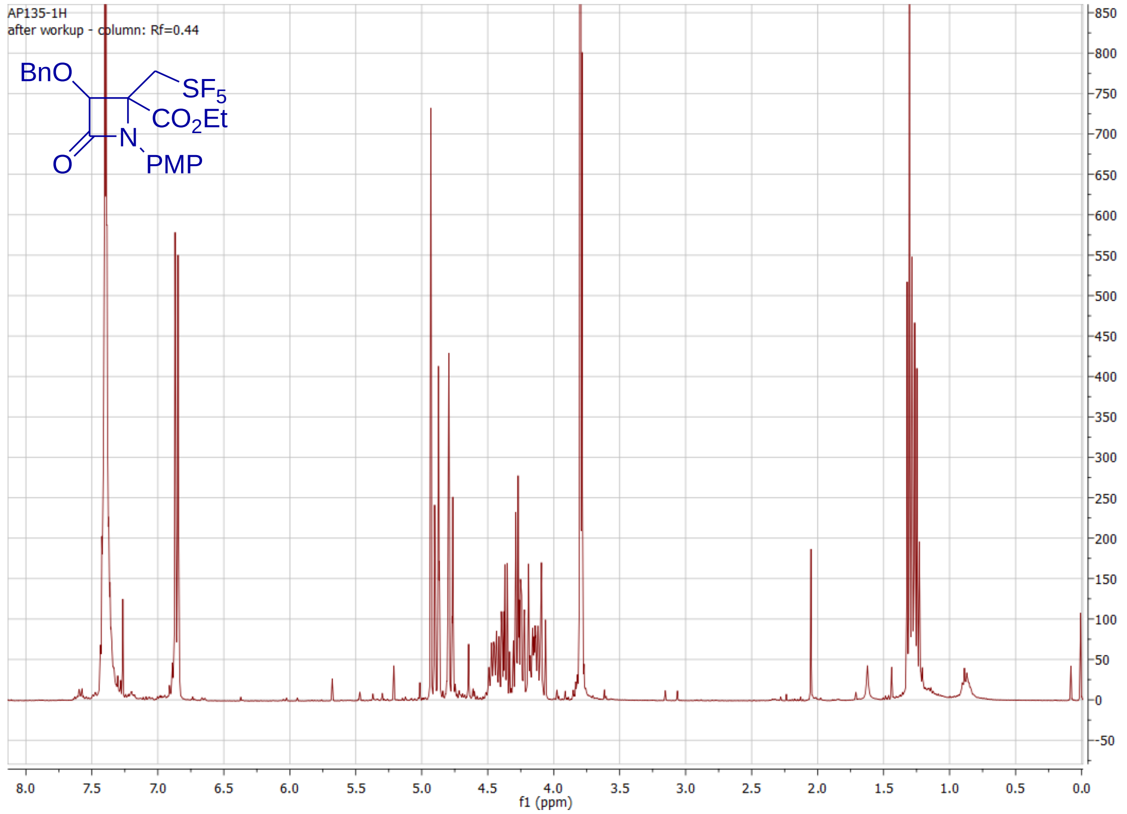
AP135-1H
after workup - column: Rf=0.44
BnO
SF5
CO2Et
N
O
PMP
f1 (ppm)

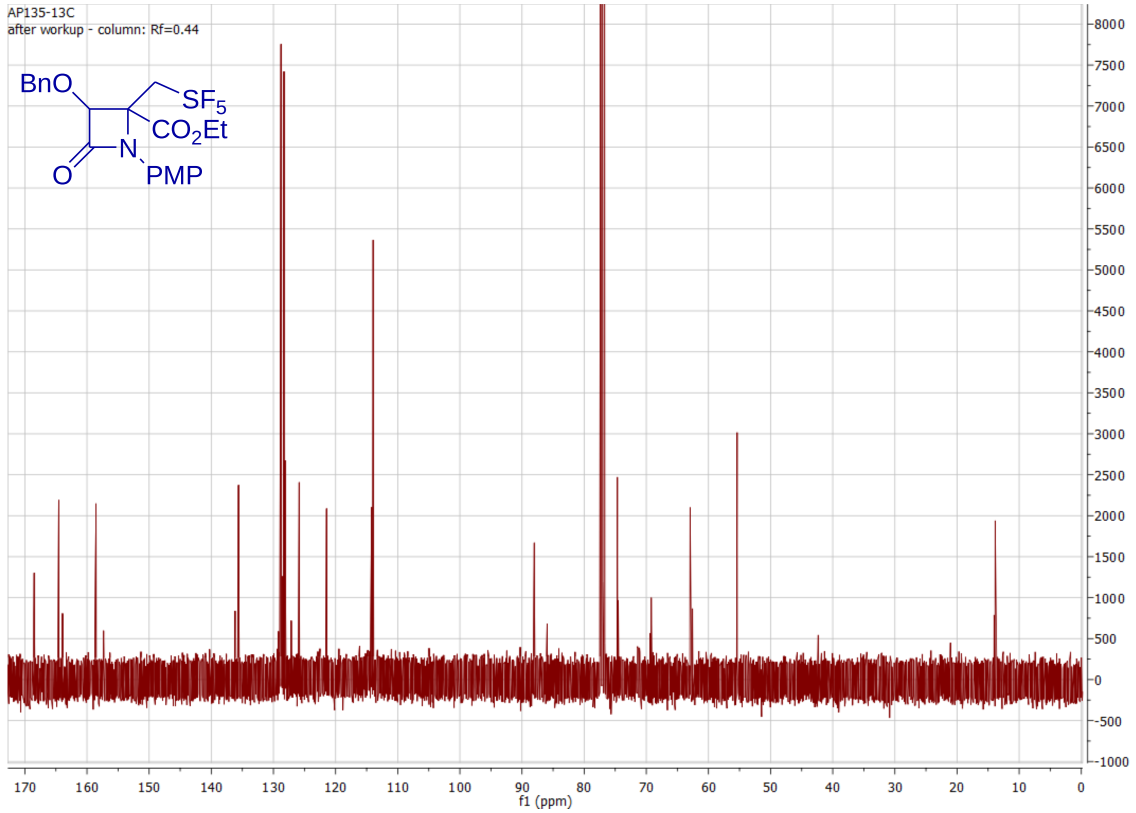
AP135-13C
after workup - column: Rf=0.44
BnO
SF5
CO2Et
N
O
PMP
f1 (ppm)

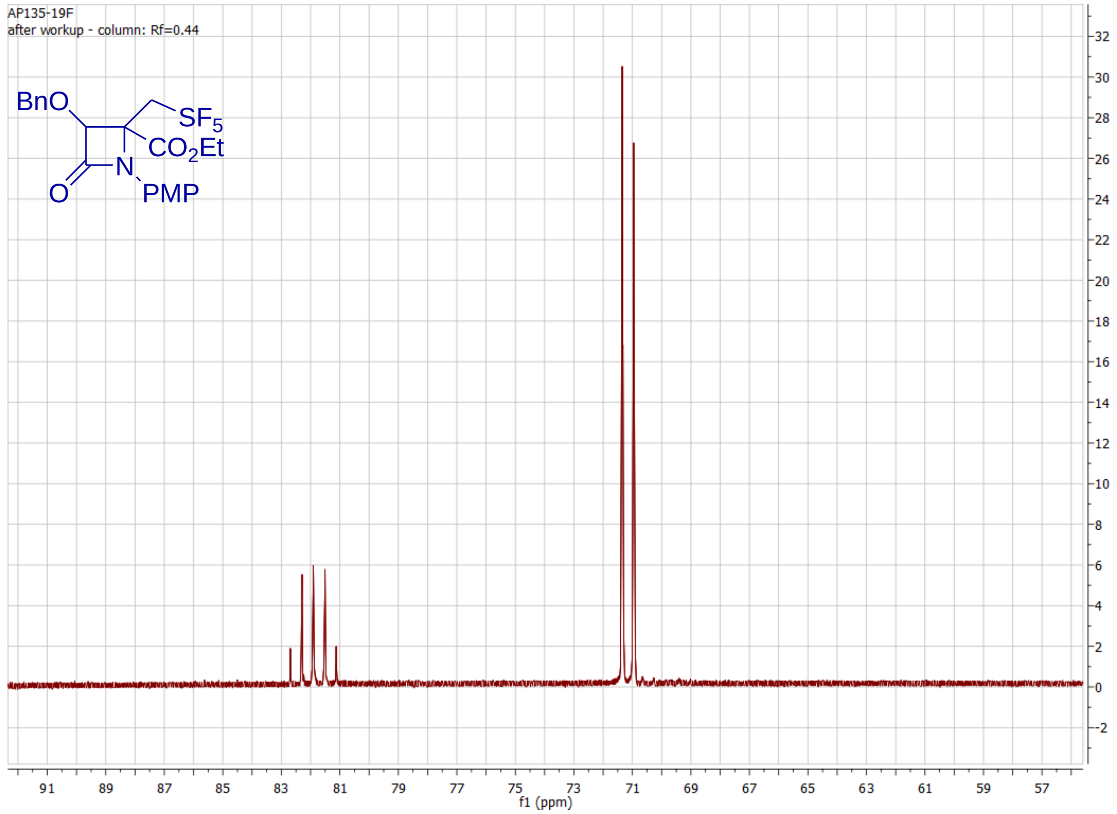

References

[1] S. C. Ngo, J.H. Lin, P. R. Savoie, E. M. Hines, K. M. Pugliese, and J. T. Welch, *Eur. J. Org. Chem.* 2012, 4902-4905.

[2] *SAINT*, version 6.02; Bruker AXS, Inc.: Madison, WI, 2001.

[3] *SADABS*; Bruker AXS, Inc.: Madison, WI, 2001.

[4] Sheldrick, G. M. *SHELXTL*, version 6.14; Bruker AXS, Inc.: Madison, WI, 2001.
